# Silver(I) Octanuclear Complexes Containing *N*′-(4-Oxotiazolidin-2-Iliden)picolinohydrazonamide and Nitrate as Bridge Ligands. An Example of Solvatomorphism?

**DOI:** 10.1021/acs.inorgchem.4c00794

**Published:** 2024-05-07

**Authors:** Isabel García-Santos, Julia Krümpelmann, Manuel Saa, Sergi Burguera, Antonio Frontera, Alfonso Castiñeiras

**Affiliations:** † Department of Inorganic Chemistry, Faculty of Pharmacy, University of Santiago de Compostela, Santiago de Compostela 15782, Spain; ‡ Department de Química, Universitat de les Illes Balears, Crta. de Valldemossa km 7.5, Palma de Mallorca 07122, Spain

## Abstract

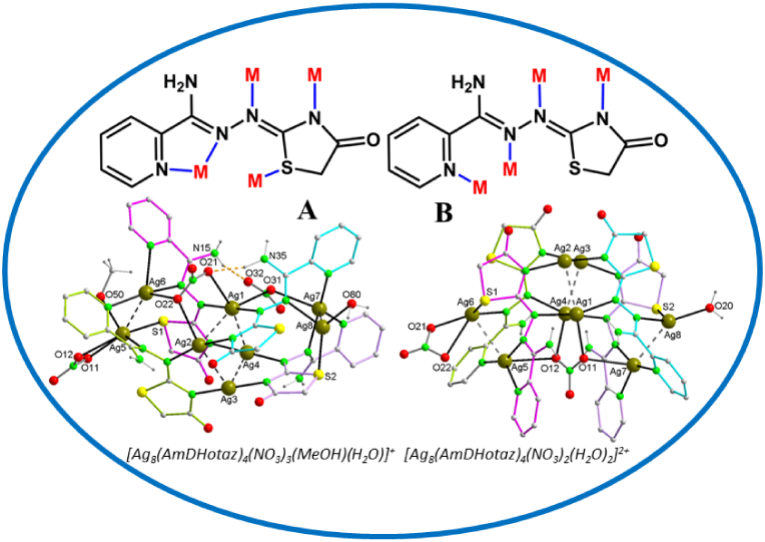

The versatile coordination chemistry of (2Z,*N*’E)-*N*′-(4-oxothiazolidin-2-ylidene)picolinohydrazonamide (HAmDHotaz) facilitated the synthesis of new complexes with different silver(I) salts. This paper describes the synthesis and characterization, through elemental analysis and spectroscopic techniques (when solubility permits), of a series of compounds that illustrate the coordinative and structural diversity achievable with the HAmDHotaz ligand. Five silver clusters containing the [Ag_8_(AmDHotaz)_4_]^4+^ nucleus were structurally analyzed by single-crystal X-ray diffraction and were found to exhibit solvomorphism. The compositions of these are [Ag_8_(AmDHotaz)_4_(NO_3_)_3_(MeOH)(H_2_O)](NO_3_)·MeOH·7.5H_2_O (**1**), {[Ag_8_(AmDHotaz)_4_(NO_3_)_3_(H_2_O)_2_](NO_3_)·9.5(H_2_O)}_n_ (**2**), {[Ag_8_(AmDHotaz)_4_(NO_3_)_3_(H_2_O)_2_](NO_3_)·11.5(H_2_O)}_n_ (**2a**), {[Ag_8_(AmDHotaz)_4_(NO_3_)_2_(H_2_O)_2_](NO_3_)(OH)·6H_2_O}_n_ (**3**), and {[Ag_8_(AmDHotaz)_4_(NO_3_)_2_(H_2_O)](NO_3_)(OH)*·*4.5H_2_O}_*n*_ (**3a**). Argentophilic interactions are present in each of the octanuclear structures, where Ag···Ag distances range from 2.828(2) to 2.986(1) Å. These distances are influenced by crystal packing, determined by the counterion and solvent molecules in the structure. In the solvatomorphs, solvent molecules were observed to be disordered. Various hydrogen-bonding interactions, such as N–H···O–N, O–H···O, N–H···O=C, C–H···O–N, and π–π stacking interactions, contribute to the crystal packing. The influence of these weak interactions on the crystal packing was further analyzed using DFT calculations and Bader’s theory of atoms-in-molecules, with a focus on argentophilic interactions and Ag···S interactions.

## Abstract 2

Five original Ag-based clusters have been constructed with *N’*-(4-oxothiazolidin-2-iliden) picolinohydrazonamide, a multidentate organic connector containing *N*. The new compounds, which exhibit argentophilic and solvatomorphism, are cross-linked via weak interactions and nitrate bridges, in one, two, or three dimensions.

## 1 Introduction

The design and synthesis of discrete multinuclear architectures and polymeric coordination networks, which utilize transition metal ions and multidentate organic ligands, have been the subject of continuous research in recent years.^1^ The use of appropriate metal cations and the selection of suitable multifunctional ligands allow the development of strategies to obtain multidimensional crystal structures.^2^ However, when cations exhibit different coordination modes and geometries, the outcomes are generally unpredictable.^3^ In this context, the controlled formation of metal–ligand complexes and their subsequent aggregation into various plurinuclear combinations is intriguing. This approach offers the potential to control concurrent interactions in creating materials with desired structures and properties,^4^ which is a key aim in both supramolecular chemistry and materials science.^5^

Unlike bonding interactions in open-shell metal atoms, metallophilic interactions are considered weak electrostatic attractive forces found between low oxidation state closed-shell [(n–1)d^10^ ns^0^] and pseudoclosed-shell [(n–1)d^8^ ns^0^] metal ions, such as Cu(I), Ag(I), Au(I), Hg(II), Pd(II), and Pt(II).^6^ Metallophilic interactions occur when two metal centers approach closely, with distances less than the sum of their van der Waals radii. This phenomenon, a type of dispersion between electron densities in large, low-valence metal ions,^7^ is influenced by factors affecting the electron density at a metal center. The presence of metallophilic interactions in solids is primarily determined through metal–metal distance analysis, with low-temperature single-crystal X-ray diffraction being an effective method for evaluating these interactions.^8^

The preparation of nanoscale silver clusters, their structural characterization by means of X-ray diffraction of single crystals at low temperature, and the different physicochemical properties such as catalysis, chirality, electrochemistry, and luminescence, for their possible application as catalysts or in optoelectronics, bioimaging, and photochemistry,^9^ has been the subject of intense study for years. Many silver clusters have been obtained by combining the elements silver and sulfur.^7^ Others are compounds that consist of a nucleus based on a metal chalcogenide surrounded by tertiary phosphane ligands or bidentate phosphanes.^10,11^ Numerous atomic-precision coinage metal nanoclusters with thiolates, phosphines, and alkynes as protective ligands have also been reported, especially among the family of silver nanoclusters.^12^ On the other hand, recent research has indicated that anion templates such as sulfide, halogen, and oxoanions (SO_4_^2–^, NO_3_^–^, CO_3_^2–^, CrO_4_^2–^, MoO_4_^2–^, and polyoxometalates) play a vital role in the synthesis of silver clusters of high nuclearity.^13^ Within the family of silver nanoclusters, there is a subgroup that presents hierarchical core–shell structures, in whose formation the growth of the metallic core is a kinetically controlled process followed by the encapsulation of the outer silver shell to finally give a thermodynamically stable product.^14^ Although many high-nuclear silver nanoclusters have been isolated with atomic precision, a systematic synthetic strategy for the controllable assembly of high-nuclear silver clusters remains less developed. Therefore, controlling the nucleation and the subsequent growth process still remains a nontrivial challenge in the field of silver nanoclusters.^15^

Silver(I), with coordination numbers ranging from 2 to 9, has been employed to construct numerous geometrically varied coordination polymers and discrete multinuclear complexes.^3,16^ Furthermore, Ag(I) can form Ag···Ag bonding interactions due to argentophilicity, useful for controlling supramolecular architectures and dimensionality.^17^ As a soft acid, silver(I) tends to coordinate with soft bases, such as ligands containing sulfur and nitrogen atoms.^18^ Thiazolidine-4-one is an example of such ligands,^19^ prominent as building blocks in various pharmaceutical agents and biologically active products. Over the past two decades, they have garnered attention for their presence in biologically active compounds, exhibiting a wide range of biological activity and therapeutic properties.^20,21^

However, if the different crystal structures are the result of a hydration or solvation process, such a phenomenon is called pseudopolymorphism or solvatomorphism and is defined as the ability of a substance to form different unit cells, which differ in their elemental composition as a consequence of the inclusion of one or more solvent molecules.^22^ Solvatomorphism is also associated with nucleation, conformational flexibility, orientation, crystalline packing of solid-phase molecules, as well as with the kinetics of reconstructing solvation layers and relaxing defects in newly created crystals.^23,24^ These differences in solvent molecules, in the case of materials such as dyes, pigments, and optical materials, can modulate supramolecular assembly resulting in changes in the energy gap band, as well as variations in color.^25,26^ Certain solvates can also be used for the storage of gases such as H_2_, natural gas, and atmospheric CO_2_, and sometimes exhibit unusual thermal properties.^27,28^ But where research on new solvomorphs has been extensively developed has been in the pharmaceutical sciences, focusing attention on the industrial level, since the inclusion of one or more solvent molecules in a crystalline structure of an active pharmaceutical ingredient (API), giving rise to different forms of solid state, affects its macroscopic properties as well as its pharmacokinetic and pharmacodynamic properties.^29^ Although the existence of organic solvents in therapeutic substances can significantly increase the toxicity of solvatomorphs, it could still be of great value to its research potential. On the other hand, the importance of solvatomorphism is also reflected in its potential contributions of new polymorphic forms obtained through solvent removal.^30^

**Chart 1:**
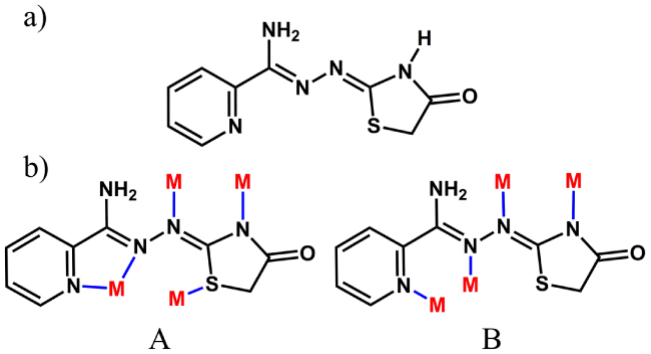
a) (2Z,*N*’E)-*N*′-(4-oxothiazolidin-2-ylidene)picolinohydrazonamide (HAmDHotaz), b) Coordination Modes of the *N*′-(4-Oxothiazolidine-2-ylidene) Picolinohydrazonamide Ligands in Silver Complexes

We have previously reported the preparation, characterization, and structural features of (2Z,*N*’E)-*N*′-(4-oxothiazolidin-2-ylidene)picolinohydrazonamide (HAmDHotaz, Chart 1) and its palladium(II) and platinum(II) complexes.^31^ Herein, we report the synthesis, crystal structures, and spectroscopic characterization of complexes of HAmDHotaz with different silver salts. The existence of noncovalent Ag···S interactions, in comparison to coordination bonds and metallophilic (Ag···Ag) interactions, are analyzed using DFT calculations and the topology of the electron density.

## 2 Experimental Section

### 2.1 Materials and General Methods

All reagents and solvents were commercially available except for HAmDHotaz, which was prepared as reported previously.^31^ Elemental analyses were performed with a Carlos Erba 1108 microanalyzer. ^1^H, ^13^C NMR spectra were obtained as DMSO-*d*_6_ solutions with a Bruker AMX 300 spectrometer. IR spectra were recorded as KBr disks (4000–400 cm^–1^) and polyethylene-sandwiched Nujol mulls (500–100 cm^–1^) with a Bruker IFS-66v spectrometer. Mass spectra were obtained in a BIOTOF II API 4000 spectrometer for ESI.

### 2.2 Synthesis of [Ag_8_(AmDHotaz)_4_(NO_3_)_3_(MeOH)(H_2_O)](NO_3_)·MeOH·7.5H_2_O (1)

A solution of AgNO_3_ (36 mg, 0.2 mmol) in H_2_O (3 mL) was added to a solution of HAmDHotaz (25 mg, 0.1 mmol) in MeOH (7 mL) under stirring for 15 min. The clear reaction mixture was kept in the dark and was allowed to evaporate and slow evaporation of this solution resulted in the colorless single crystals of [[Ag_8_(AmDHotaz)_4_(NO_3_)_3_(MeOH)(H_2_O)](NO_3_)·MeOH·7.5H_2_O (**1**) suitable for an X-ray analysis.

Yield **1**: 50% (0.030g). Mp 207 °C. Elemental analysis: Found: C, 19.3; H, 2.1; N, 14.6; S, 5.4. Calc. for C_38_H_57_Ag_8_N_24_O_26.5_S_4_: C, 20.1; H, 2.5; N, 14.8; S, 5.6%. IR (ν_max_/cm^–1^): 3559 ν(OH), 3483–3357 ν(NH), 1706 ν(C=O), 1639, 1626 δ(NH), 1520–1589 ν(C=N+C=C), 999 ν(NN), 1384 ν(NO_3_^–^). ESI MS, *m*/*z*, assignment: 684.9, [Ag_2_(L)_2_]; 790.8, [Ag_3_(HL-2H)_2_]; 898.7, [Ag_4_(HL-2H)_2_]; 1133.8, [Ag_4_(L)_3_]; 1241.7, [Ag_5_(L)_3_]; 1476.7, [Ag_5_(L)_4_]; 1582.6, [Ag_6_(L)_2_(HL-2H)_2_]; 1690.5, [Ag_7_(L)_2_(HL-2H)_2_]; 1817.7, [Ag_6_(L)_4_(HL-2H)]; 1923.6, [Ag_7_(L)_2_(HL-2H)_3_]; 2031.5, [Ag_8_(L)_2_(HL-2H)_3_]; 2160.6, [Ag_7_(L)_6_]; 2266.5, [Ag_8_(L)_4_(HL-2H)_2_]. ^1^H NMR (DMSO-*d*_6_, ppm): 8.81 (1H, s, H1); 8.07 (2H, s, H4+H3); 7.71 (1H, s, H2); 7.58 (2H, bs, NH_2_); 3.88 (2H, s, CH_2_). ^13^C NMR (DMSO-*d*_6_, ppm): 181.21 (C=O), 176.14 (C7), 154.49 (C6), 150.19 (C5), 148.01 (C1), 138.74 (C3), 126.82 (C2), 122.70 (C4), 36.04 (CH2).

Besides, crystals of {[Ag_8_(AmDHotaz)_4_(NO_3_)_3_(H_2_O)_2_](NO_3_)·9.5(H_2_O)}_*n*_ (**2**), {[Ag_*8*_(AmDHotaz)_4_(NO_3_)_3_(H_2_O)_2_](NO_3_)·11.5(H_2_O)}_*n*_ (**2a**), {[Ag_8_(AmDHotaz)_4_(NO_3_)_2_(H_2_O)_2_](NO_3_)(OH)*·*6H_2_O}_*n*_ (**3**), and {[Ag_8_(AmDHotaz)_4_(NO_3_)_2_(H_2_O)](NO_3_)(OH)·4.5H_2_O}_*n*_ (**3a**) were also obtained when the reaction was repeated.

### 2.3 X-Ray Crystallography

Diffraction data were obtained at 100.0(1) K on a Bruker X8 KappaAPEXII diffractometer from yellow crystals of **1**–**3** mounted on glass fibers. Graphite monochromated MoK(α) radiation (λ = 0.71073 Å) was used throughout. The data were processed with APEX2^32^ and corrected for absorption using SADABS.^33^ The structures were solved by direct methods^34^ and refined by full-matrix least-squares techniques against *F*^2^.^34^ Positional and anisotropic atomic displacement parameters were refined for all non-hydrogen atoms. Hydrogen atoms attached to carbon were located in difference Fourier maps or placed in geometrically idealized positions. The O–H and N–H hydrogen atoms were located from difference maps. All hydrogen atoms were refined using a riding model. Molecular graphics were generated with DIAMOND.^35^ Details on the particularity in the resolution of each structure are collected in Supporting Information, and the crystal data, experimental details, and refinement results are summarized in Table 1.

**Table 1:** Crystal Data and Structure Refinement for Compounds 1 to 3

compound	1	2	2a	3	3a
empirical formula	C_38_H_57_Ag_8_N_24_O_26.5_S_4_	C_36_H_55_Ag_8_N_24_O_27.5_S_4_	C_36_H_59_Ag_8_N_24_O_29.5_S_4_	C_36_H_50_Ag_8_N_23_O_22_S_4_	C_36_H_45_Ag_8_N_23_O_19.5_S_4_
formula weight	2265.27	2255.24	2291.27	2148.19	2103.15
temperature/K	100(2)	100(2)	100(2)	100(2)	100(2)
wavelength/Å	0.71073	0.71073	0.71073	0.71073	0.71073
crystal system	triclinic	triclinic	triclinic	orthorhombic	orthorhombic
space group	*P*	*P*	*P*	*Pna*2_1_	*Pna*2_1_
unit cell dimensions					
*a*/Å	14.528(4)	14.4297(5)	14.5438(4)	30.9867(12)	30.9915(12)
*b*/Å	14.826(4)	14.8045(5)	14.9365(4)	15.0720(5)	15.0756(5)
*c*/Å	16.510(4)	16.5989(6	16.6151(4)	13.5982(5)	13.6003(5)
α/°	78.840(4)	78.355(2)	78.8090(10)	90	90
β/°	87.479(4)	87.561(2)	88.3910(10)	90	90
γ/°	77.325(4)	75.984(2)	75.023(2)	90	90
volume/Å^–3^	3403.9(16)	3369.5(2)	3419.52(16)	6350.8(4)	6354.3(4)
*Z*	2	2	2	4	4
calc. density/Mg/m^3^	2.210	2.223	2.225	2.247	2.198
absorp. coefc./mm^–1^	2.469	2.494	2.462	2.634	2.627
*F*(000)	2210	2198	2238	4172	4072
crystal size	0.20 × 0.08 × 0.05	0.16 × 0.11 × 0.04	0.08 × 0.08 × 0.06	0.16 × 0.05 × 0.05	0.16 × 0.05 × 0.05
θ range/°	1.719–25.681	1.455–27.877	1.713–25.682	1.885–30.533	1.885–30.516
limiting indices/*h,k,l*	–17/17,–17/18,0/20	–18/18,–18/19,0/21	–17/17,–17/18,0/20	0/44,0/21,–19/19	–44/43,–21/21,–19/19
refl. collect/unique	38502/12898	86514/16007	78787/12884	130098/19259	128576/19312
*R*_int_	0.0379	0.0492	0.0542	0.0533	0.0674
absorp. correct.	multiscan	multiscan	multiscan	multiscan	multiscan
max. /min transm.	1.000/0.639	1.000/0.845	1.000/0.713	1.000/0.735	1.000/0.873
data/rest/parameters	12898/30/875	16007/24/901	12884//96/909	19259/73/819	19312/19/823
goodness-of-fit on *F*^2^	1.046	1.027	0.991	1.037	1.057
final *R* indices	*R*_1_ = 0.0468, *wR*_2_ = 0.1046	*R*_1_ = 0.0354, *wR*_2_ = 0.0844	*R*_1_ = 0.0624, *wR*_2_ = 0.1385	*R*_1_*=* 0.0688, *wR*_2_ *=* 0.1572	*R*_1_ = 0.0702, *wR*_2_ = 0.1610
*R* indices (all data)	*R*_1_ = 0.0837, *wR*_2_ = 0.1146	*R*_1_ = 0.0497, *wR*_2_ = 0.0916	*R*_1_ = 0.1217, *wR*_2_ = 0.1575	*R*_1_ = 0.0864, *wR*_2_ = 0.1651	*R*_1_ = 0.0895, *wR*_2_ = 0.1719
largest dif. peak/hole	2.296/–1.227	2.517/–1.327	1.943/–1.208	3.814/–1.840	3.612/–1.885
*CCDC number*	2321205	2321206	2321207	2321208	2321204

### 2.4 Theoretical Methods

The calculations of the noncovalent interactions were carried out using the Gaussian-16 program^37^ and the PBE0-D3/def2-TZVP level of theory.^37−39^ To evaluate the interactions in the solid state, the crystallographic coordinates have been used. The Bader’s “Atoms in molecules” theory (QTAIM)^40^ has been used to study the interactions discussed herein by means of the AIMAll calculation package.^41^ The molecular electrostatic potential surfaces (isosurface 0.002 au) have been computed using Gaussian-16 software.^36^ The NBO analysis^42^ has been performed using the NBO 7.0 program^43^ at the same level of theory.

## 3 Results and Discussion

The compounds have been characterized by X-ray diffraction, elemental analysis, FTIR, ^1^H and ^13^C NMR and ES-MS including MALDI-TOF (see Supporting Information). In the Experimental Section, an assignment of the most significant bands found in the IR spectra of the complexes is proposed. The mass spectra show a wide distribution of molecular ions. Also, it is important to note that, although the four coordinated ligands are in crystallographically unique positions with different coordination modes, which is discussed later in the description of the structures, the NMR data show a single set of resonances, indicating a dynamic equilibrium in solution. Although the *R* factors obtained for the crystal structures are very acceptable and the main body of the clusters is quite well-defined, some of the C and N atoms of the ligands have high thermal parameters, so they have been modeled including restrains of the coefficient of displacement to prevent these atoms being split into two positions. Some nitrate ions and crystallization water molecules are also affected with a considerable degree of disorder, as their anisotropic displacement thermal parameters show, and disordered atoms were refined isotropically. In these cases, hydrogen atoms of such molecules could not be located. In compounds **3** and **3a** the structure was refined as a 2-component inversion twin. There is a small component of twinning, which is seen in the diffraction images; too small to process the data, but strong enough that it generates these meaningless residual electron density remnants in the structure. The solution of the structures was extremely difficult; however, most atoms could be located without ambiguities in the Fourier difference analysis and the final results can be considered very satisfactory from the chemical and structural point of view.

### 3.1 Descriptions of the Structures

#### 3.1.1 Structure of [Ag_8_(AmDHotaz)_4_(NO_3_)_3_(MeOH)(H_2_O)](NO_3_)·MeOH·7.5H_2_O (**1**)

**Figure 1:**
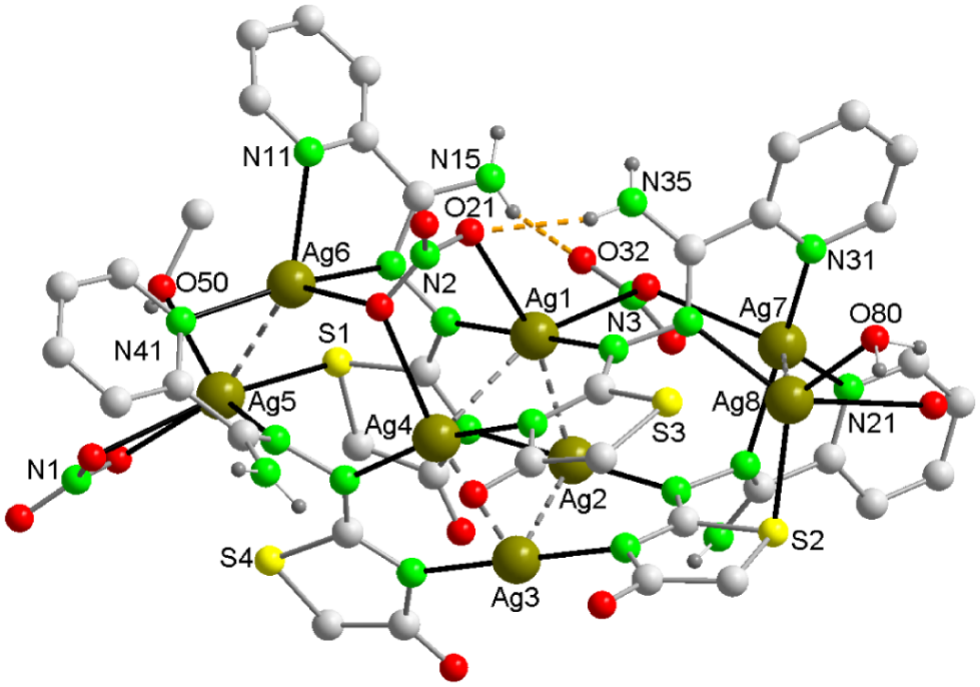
In the crystal structure of **1**, Ag_4_ and Ag_2_ aggregates are interconnected through Ag–N, Ag–O and Ag–S bonds to give the [Ag_8_(AmDHotaz)_4_(NO_3_)_3_(MeOH)(H_2_O)]^+^ cation. Color code: Ag, dark yellow; S, yellow; C, pale gray; O, red; N, bright green, H, dark gray.

**Figure 2:**
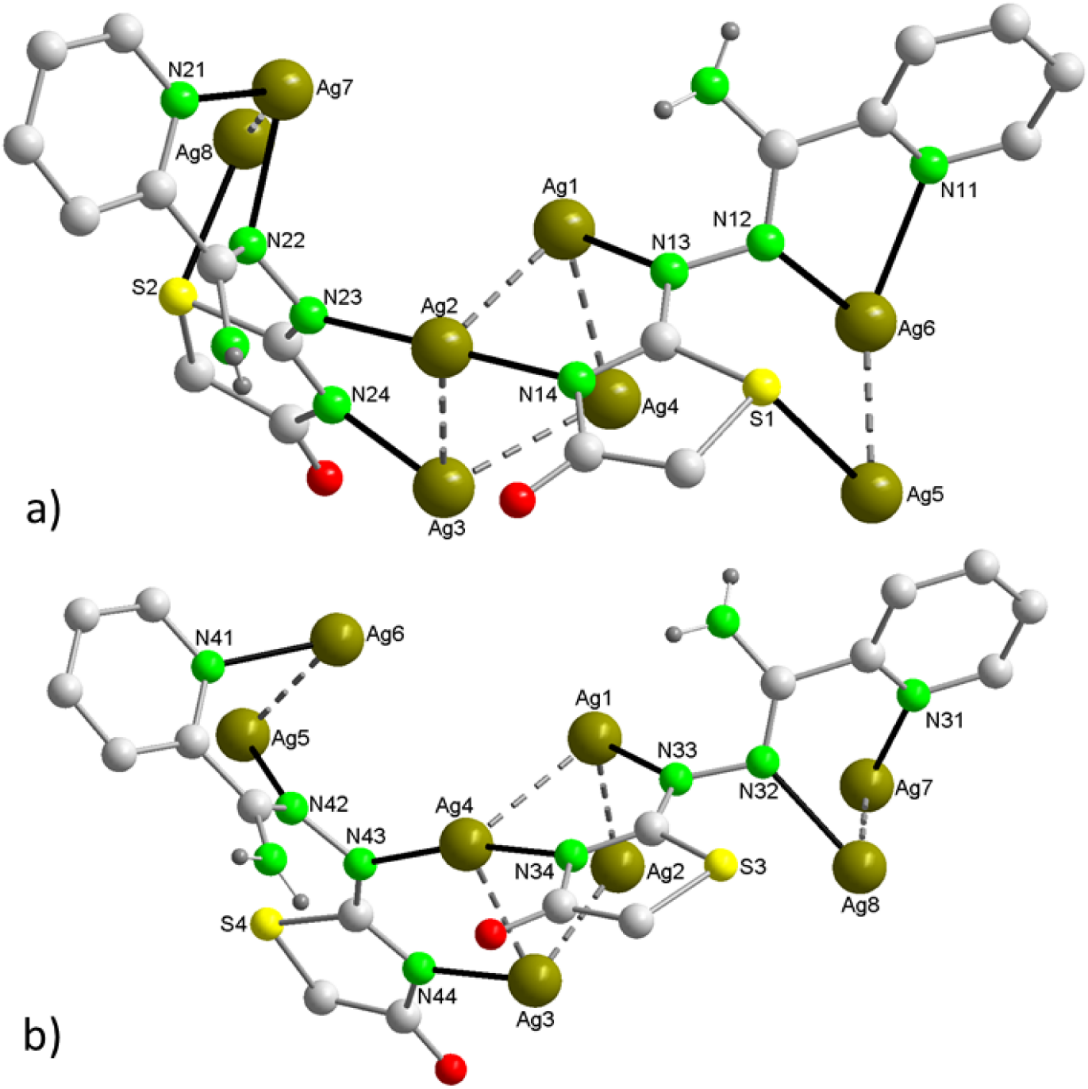
Coordination mode of the (AmDHotaz)^−^ anions in **1**, showing the bridge ligand character between four Ag^+^ ions. a) Mode A and b) Mode B.

The single crystal X-ray analysis revealed that the asymmetric unit of compound **1** comprises the species indicated by its molecular formula. This includes a cation composed of eight silver atoms coordinated by four ligands [AmDHotaz]^−^, three NO_3_^–^ ions, a methanol molecule, and a water molecule (Figure 1). Additionally, there is an anion of NO_3_^–^ and 7.5 water molecules of solvation. In the cation’s structure, a cluster of four Ag(I) atoms is approximately at the center, displaying a slightly distorted planar square geometry. This distortion is evident in the dihedral angles between the planes formed by every three contiguous Ag atoms, with values of Ag1–Ag2–Ag3/Ag3–Ag4–Ag2 = 23.48(2)° and Ag2–Ag3–Ag4/Ag4–Ag1–Ag2 = 24.56(2)°, compared to the 0° of an ideal square. The alternating Ag atoms are displaced approximately 0.22 Å from the median plane’s opposite faces. In Ag4, the Ag–Ag distances range between 2.898(1) and 2.986(1) Å (Table 2), which is between the sum of two van der Waals radii (3.44 Å) and the distance of 2.53 Å present in Ag_2_, but close to the 2.89 Å value in metallic silver crystals. This indicates significant argentophilic interactions between Ag(I) ions in this cluster.^17,44,45^ The Ag–Ag–Ag angles, varying from 85.57(3) to 92.43(3)° against the ideal 90° of a square, further quantify the cluster’s distortion. Flanking the Ag_4_ cluster and at distances of 4.227(2) and 4.067(2) Å, there are separate dinuclear clusters with Ag5–Ag6 distances of 2.921(1) Å and Ag7–Ag8 of 2.931(1) Å (Figure 1). The eight Ag^+^ ions are connected through four monoanionic organic ligands, which, from a coordination perspective, can be differentiated into two types (Chart 1). Type A ligands coordinate an Ag^+^ ion of a dinuclear cluster through the imino-pyridine group, forming a flat five-membered chelate C_2_N_2_Ag, and the other Ag^+^ ion of the same cluster through the sulfur atom, forming a nonplanar six-membered metalacycle CN_2_SAg_2_. At the other end, they coordinate two contiguous Ag^+^ ions of the Ag_4_ cluster (Ag1–Ag2 or Ag2–Ag3) through the imino-thiazolidine group, with each nitrogen atom bonded to a different Ag, forming a planar five-membered metalacycle CN_2_Ag_2_ (Figure 2a). Type B ligands, at one end, also coordinate two contiguous ions of the tetranuclear cluster (e.g., Ag1–Ag2/Ag1-Ag4), while at the other end, the two nitrogen atoms of the imino-pyridine group each coordinate to an Ag^+^ ion of the second Ag_2_ cluster, forming a nonplanar six-membered metalacycle C_2_N_2_Ag_2_ (Figure 2b). In the Ag_2_ dinuclear clusters, the Ag5–S1 and Ag8–S2 binding distances (Table 2) fall within the expected range for the mean Ag–S bond length in silver complexes with sulfur atom donor ligands. Additionally, these same silver(I) ions also engage in weak contacts with the S4 and S3 atoms of the thiazolin-one rings of type B ligands, respectively, which are very close to the maximum bond distance of 3.01 Å accepted for Ag–S bonds (Figure 2).

**Table 2:** A Summary of Important Bond Length Data (Å) for Synthesized Clusters

1		2	2a		3	3a
**Ag–Ag (CCDC av. 3.005 Å); (ΣvdW= 3.44Å)**						
Ag(1)–Ag(2)	2.9735(11)	2.9845(5)	2.9817(11)	Ag(1)–Ag(2)	2.8406(15)	2.8428(16)
Ag(2)–Ag(3)	2.8981(10)	2.9123(5)	2.9138(12)	Ag(2)–Ag(3)	3.0515(17)	3.0507(17)
Ag(3)–Ag(4)	2.9497(11)	2.9470(5)	2.9369(11)	Ag(3)–Ag(4)	2.8277(15)	2.8280(16)
Ag(1)–Ag(4)	2.9858(10)	2.9834(5)	2.9862(11)	Ag(1)–Ag(4)	3.4237(15)	3.4253(16)
Ag(5)–Ag(6)	2.9212(10)	2.9457(5)	2.9470(12)	Ag(5)–Ag(6)	2.9702(18)	2.9714(19)
Ag(7)–Ag(8)	2.9307(11)	2.9046(6)	2.8854(15)	Ag(7)–Ag(8)	2.9205(17)	2.9213(18)
**Ag–S (CCDC av. 2.555 Å); (ΣvdW = 3.52Å)**						
Ag(5)–S(1)	2.677(2)	2.7229(11)	2.722(3)	Ag(5)–S(1)	2.564(4)	2.564(4)
Ag(5)–S(4)	3.082(2)	3.1020(11)	3.117(3)	Ag(5)–S(4)	3.323(4)	3.319(4)
Ag(8)–S(2)	2.495(2)	2.5104(13)	2.507(3)	Ag(8)–S(2)	2.606(4)	2.607(4)
Ag(8)–S(3)	3.107(2)	3.1688(15)	3.191(3)	Ag(8)–S(3)	3.113(4)	3.117(4)
**Ag–O (CCDC av. 2.446 Å); (ΣvdW = 3.24Å)**						
Ag(1)–O(31)	2.725(5)	2.684(4)^*a*^	2.693(7)			-
Ag(1)–O(21)	2.750(5)	2.769(3)	2.770(7)	Ag(2)–O(21)	2.433(11)	2.437(11)
Ag(4)–O(22)	2.652(5)	2.600(3)	2.571(7)	Ag(3)–O(22)	2.455(10)	2.448(11)
Ag(5)–O(50)	2.418(5)	2.348(3)	2.348(7)	Ag(5)-O(11B)		2.63(3)
Ag(5)–O(11)	2.516(6)	2.507(4)	2.509(8)	Ag(5)-O(11A)	2.30(2)	2.25(2)
Ag(5)–O(12)	2.668(6)	2.789(4)	2.774(8)	Ag(5)-O(12A)/O(50)	2.80(4)	2.99(3)
Ag(6)–O(22)	2.677(5)	2.710(3)	2.701(7)	Ag(6)–O(21)	2.794(11)	2.795(11)
Ag(7)–O(31)	2.703(6)	2.713(4)^*a*^	2.669(7)	Ag(7)–O(22)	2.785(10)	2.789(11)
Ag(8)–O(13)	-	2.846(4)^*b*^	2.870(8)^*b*^	Ag(8)-O(13A/B)	2.747(15)^*a*^	2.64(4)/2.36(4)^*a*^
Ag(8)–O(80)	2.262(6)	2.297(5)	2.259(10)	Ag(8)-O(80A/B)	2.31(2)	2.27(2)/2.83(3)^*a*^
**Ag–N (CCDC av. 2.267 Å); (ΣvdW = 3.27Å)**						
Ag(1)–N(13)	2.123(6)	2.128(4)	2.116(9)	Ag(1)–N(44)	2.802(12)	2.103(12)
Ag(1)–N(33)	2.141(6)	2.122(4)	2.133(10)	Ag(1)–N(24)	2.097(11)	2.095(11)
Ag(2)–N(14)	2.098(6)	2.118(4)	2.115(8)	Ag(2)–N(23)	2.197(11)	2.181(12)
Ag(2)–N(23)	2.131(6)	2.137(4)	2.151(9)	Ag(2)–N(43)	2.206(13)	2.187(13)
Ag(3)–N(24)	2.087(6)	2.090(4)	2.087(8)	Ag(3)–N(33)	2.170(11)	2.183(11)
Ag(3)–N(44)	2.097(6)	2.090(4)	2.098(8)	Ag(3)–N(13)	2.199(10)	2.205(11)
Ag(4)–N(34)	2.140(6)	2.143(4)	2.140(9)	Ag(4)–N(34)	2.088(13)	2.094(12)
Ag(4)–N(43)	2.169(6)	2.154(4)	2.135(8)	Ag(4)–N(14)	2.098(11)	2.106(11)
Ag(5)–N(42)	2.342(6)	2.321(4)	2.303(9)	Ag(5)–N(42)	2.244(12)	2.294(13)
Ag(6)–N(41)	2.233(6)	2.239(4)	2.238(8)	Ag(6)–N(41)	2.217(14)	2.211(14)
Ag(6)–N(12)	2.244(6)	2.268(4)	2.286(8)	Ag(6)–N(12)	2.309(12)	2.294(13)
Ag(6)–N(11)	2.396(6)	2.366(4)	2.370(9)	Ag(6)–N(11)	2.334(13)	2.338(13)
Ag(7)–N(31)	2.218(6)	2.236(4)	2.228(9)	Ag(7)–N(31)	2.184(12)	2.181(12)
Ag(7)–N(22)	2.277(6)	2.283(4)	2.305(8)	Ag(7)–N(22)	2.237(13)	2.250(14)
Ag(7)–N(21)	2.367(6)	2.379(4)	2.361(9)	Ag(7)–N(21)	2.327(12)	2.321(12)
Ag(8)–N(32)	2.296(6)	2.303(4)	2.292(8)	Ag(8)–N(32)	2.294(12)	2.287(13)
Symmetry transformations		(*a* = −*x* + 1, −*y* + 1, −*z* + 1), (*b* = *x*, *y*, *z* + 1)	(*b* = *x*, *y*, *z* + 1)		(*a* = *x*, −1 + *y*, *z*)	(*a* = *x*, −1 + *y*, *z*)

For the four ligands, the Ag–N bond lengths in the complex align with values found in complexes of silver(I) with nitrogen atom donors. However, a more detailed analysis of the Ag–N bonds (Table 2) reveals that the bonds with the nitrogen atoms of the imino-pyridine group, N_py_ and N_az_, are approximately 8% longer than those between the Ag^+^ ions and the nitrogen atoms of the imino-thiazolidine group, N_taz_. Moreover, in the ligands that also coordinate to silver(I) through the sulfur atom, the Ag–N_py_ bond is longer than the Ag–N_az_ bond, while in those without such coordination, the reverse is true. This observation, consistent with behavior in complexes of AmDHotaz and related Fe(III), Pd(II), Pt(II), Cu(II), and Zn(II) ligands,^31,46−48^ where it acts as either a flat tridentate ligand through N_py_, N_az_, and N_taz_, or as a bidentate ligand through N_py_ and N_az_, indicates a strengthening of the Ag–N_az_ bond, most likely due to the chelate effect of two fused rings.

The nitrogen atoms of the imino-thiazolidine group, N_hy_ and N_taz_, exhibit stronger binding to silver(I), probably due to the formation of flat five-membered chelates involving two Ag^+^ ions. This might also explain why the Ag–N_az_ bond is stronger than the Ag–N_py_ bond, although in the latter case, the six-membered metalacycle is not flat, leading to a weaker Ag–N_az_ bond. The Ag–O lengths (Table 2) align with values found in the CSD for Ag–O_Nitrate_ lengths of different denticity.^49^ It is well-established that the nitrate ion, as a ligand, coordinates numerous metal ions through its oxygen atoms in approximately 30 different ways, either as a terminal or bridging ligand with varying denticity.^50^

**Figure 3:**
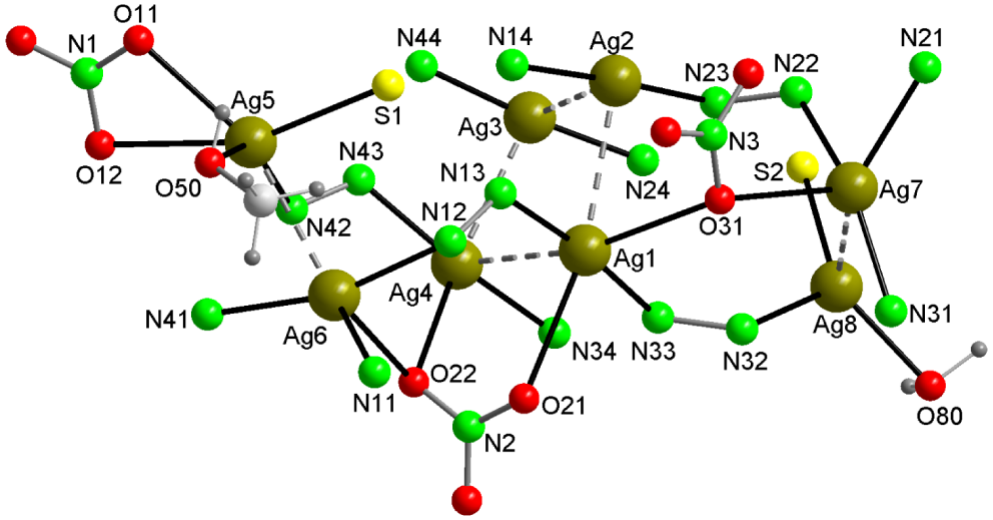
The coordination environment of the Ag(I) ions in 1, including Ag···Ag interactions (gray dashed lines). All irrelevant atoms and hydrogen atoms are omitted for clarity.

In the complex, there are three distinct modes of coordination. The first mode involves nitrate N1, acting as a simple bidentate or symmetric bidentate terminal, which binds to the Ag5 ion via the O11 and O12 atoms. The second mode is exhibited by nitrate N2, functioning as a bidentate, monodentate bridge. It coordinates the Ag1, Ag4, and Ag6 ions through the O21 and O22 atoms. The third mode involves nitrate N3, operating as a bidentate bridge, coordinating the Ag1 and Ag7 ions through the O31 atom. Additionally, Ag5 is coordinated by a methanol molecule and Ag8 by a water molecule, with both distances falling within expected ranges. Considering the coordinating ligands described above, the numbers and coordination geometries adopted by the eight silver(I) ions are quite diverse (Figure 3).

The coordination environment of Ag1 and Ag6 can be described as a seesaw or sawhorse geometry with angles of 172.9(2)° (N13–Ag1–N33) and 117.1(2)° (O21–Ag1–O31) for Ag1, and 164.3(2)° (N12–Ag6–N41) and 97.1(1)° (N11–Ag6–O22) for Ag6. Ag2 and Ag3 are situated in an almost linear geometry, with angles of 176.6(2)° and 168.8(2)°, respectively, and each is coordinated by two nitrogen atoms from different AmDHotaz ligands. Ag4 adopts an almost T-shaped geometry with two nitrogen atoms from two AmDHotaz ligands in the horizontal direction and an oxygen atom from a nitrate in the axial direction to form a T-shaped unit. Ag5′s coordination geometry is a distorted tetrahedron, with the distortion quantified by the τ4 parameter introduced by Houser et al.,^51^ which in this case is 0.58 (τ_4_ = 1 for a perfect tetrahedron). The coordination includes a nitrate ion as a nearly symmetric chelating bidentate ligand, a sulfur atom from one AmDHotaz ligand, a nitrogen atom from a second AmDHotaz, and an oxygen atom from a methanol molecule. Ag7 exhibits a distorted tetrahedral geometry with a τ_4_ of 0.65, coordinated by two nitrogen atoms from an AmDHotaz, a nitrogen atom from another AmDHotaz ligand, and an oxygen atom from a nitrate. The Ag8 coordination environment is Y-shaped, comprising a sulfur atom from an AmDHotaz ligand, a nitrogen atom from another ligand, and an oxygen atom from a water molecule, with a large bond angle of 134.4(1)°.

From a classical hydrogen bonding perspective, in the cation of **1**, each AmDHotaz ligand contains two N–H bonds as donors and one oxygen atom as an acceptor. Additionally, the coordinated water molecule offers two donor O–H bonds and an acceptor oxygen atom, and the methanol molecule includes a donor O–H bond and an acceptor oxygen atom. The oxygen atoms of the coordinated nitrates must also be taken into account. Considering that the compound also contains 7.5 water molecules of solvation, it is evident that the crystal packing in **1** is governed by numerous hydrogen bonds (Table S1). In the cation [Ag_8_(AmDHotaz)_4_(NO_3_)_3_(MeOH)(H_2_O)]^+^, two strong hydrogen bonds involve an NH bond from each of the two amine groups N15 and N35 as donors, and the oxygen atoms O32 and O21, respectively, of two coordinated nitrates, as proton acceptors (Figure S7a, Table S1). Furthermore, the separation of 3.25 Å between the Ag8 cation and the Cg5 centroid of the thiazolidine ring S2 falls within the range of 2.89–3.37 Å, indicative of an intracationic silver(I)-π interaction (Figure S7b, Table S2).^52^

The cations in the complex are interconnected through intermolecular “head-to-tail” OH···O interactions. These involve the oxygen atom O1 of the water molecule coordinated to Ag8 and the oxygen atom O12 of the nitrate ion coordinated to Ag5 of the nearest neighboring cation. There are also interactions between the oxygen atom O50 of the methanol molecule coordinated to Ag5 and the oxygen atom O33 of the nitrate bridging the Ag1 and Ag7 ions of an adjacent cation. This results in an infinite two-dimensional network propagating along the “*c*″ axis. The distances H1B···O12^*b*^ (*b*: *x*, *y*, *z* + 1) at 1.85 Å and H50A···O33^*c*^ (*c*: −*x*, *–y*, *–z* + 1) at 1.93 Å suggest these interactions are very strong. Moreover, cations and nitrate anions are connected by a strong hydrogen bond between the second OH bond of the coordinated water molecule and one of the oxygen atoms O41 of the nitrate, with a distance H1A···O41^*a*^ (*a*: 1 – *x*, 1 – *y*, −*z*) of 1.88 Å (Figure S7a, Table S1). The cation also displays aromatic ring-aromatic ring interactions between the pyridine units of different cations.

As depicted in Figure S8, the reference cation interacts with the nearest neighboring cation (symmetry transformation: −*x*, 1 – *y*, 1 – *z*) via stacking interactions, with a centroid-centroid distance of 3.46 Å (Table S2). These interactions reinforce the previously described two-dimensional network. An intercationic silver(I)-aromatic ring interaction also contributes to the stability of the cation chains. The 3.46 Å distance between the centroid of the S4 thiazolidine ring of the AmDHotaz ligand and the Ag3^*f*^ cation (*f*: 1 – *x*, −*y*, 1 – *z*) is slightly beyond the typical range and suggests a weak Ag-π interaction.^53^ Furthermore, weak CH···ring interactions between neighboring cations are observed, involving the carbon α of the pyridine ring, C31, and the four-membered chelate ring formed by the Ag5 atom and the coordinated nitrate (Table S2). Overall, the complex cation chains extend along the crystallographic “c″ axis, forming channels in their widest cavity, where the nitrate counterion, a methanol molecule, and the crystallization water molecules are located. The minimum internal dimensions of this cavity are approximately 5.9 × 7.5 × 12.0 Å^3^ (H···H measurements), equating to an approximate volume of 789 Å^3^. These species are connected to the cations by hydrogen bonds O–H···O, forming two-dimensional networks (Figure S9, Table S1).

#### 3.1.2 Structures of {[Ag_8_(AmDHotaz)_4_(NO_3_)_3_(H_2_O)_2_](NO_3_)·9.5(H_2_O)}_n_ (2) and {[Ag_8_(AmDHotaz)_4_(NO_3_)_3_(H_2_O)_2_](NO_3_)·11.5(H_2_O)}_n_ (2a)

**Figure 4:**
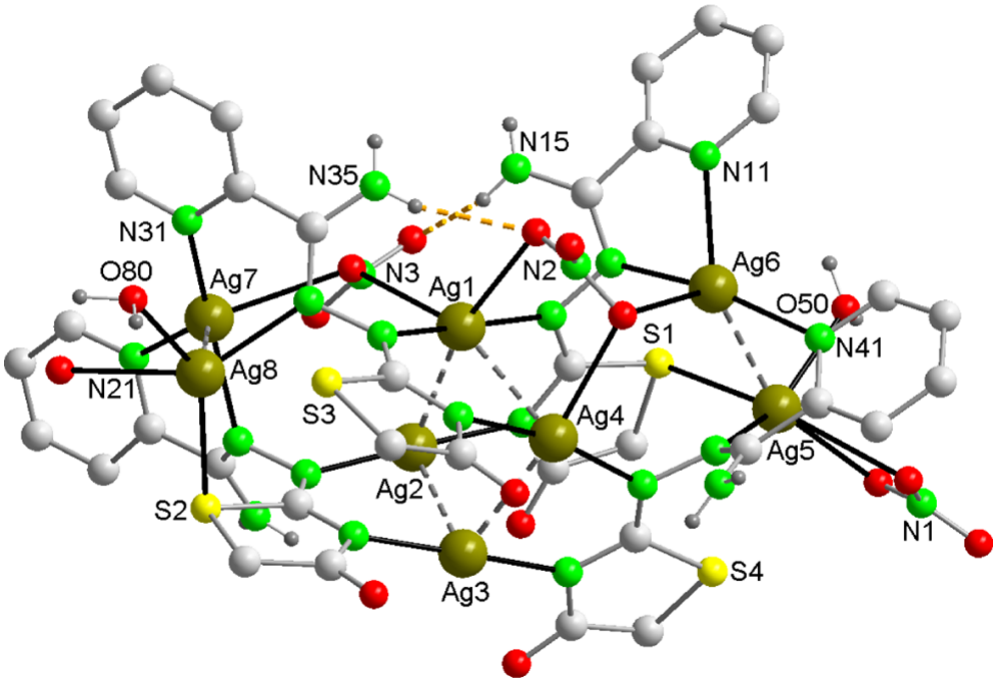
View of [Ag_8_(AmDHotaz)_4_(NO_3_)_3_(H_2_O)_2_]^+^ cation in the asymmetric unit of **2** where the Ag_4_ and Ag_2_ aggregates are interconnected through Ag–N, Ag–O and Ag–S. Orange dashed lines indicate hydrogen bonds. Color code: Ag, dark yellow; S, yellow; C, pale gray; O, red; N, bright green, H, dark gray.

Both compounds are isostructural, mainly differing in the number of crystallization molecules. Consequently, the data from the structure solution of **2**, which has higher precision, will be primarily referenced. In many ways, the structures of compounds **1** and **2** can be considered configurationally isotopic, sharing the same space group, similar unit cell dimensions, and comparable positional coordinates.^54^ However, they differ in the number of atoms in the unit cell. The methanol molecules in **1** (both coordinated and crystallization) have been replaced by water molecules in **2**. Also, compound **2** contains a greater number of crystallization water molecules. Like **1**, the asymmetric unit of **2** (Figure 4) consists of eight Ag^+^ ions, four molecules of the deprotonated ligand Am4DHotaz^–^, four nitrate ions, and 13 and a half water molecules, forming a cation [Ag_8_(AmDHotaz)_4_(NO_3_)_3_(H_2_O)_2_]^+^, a NO_3_^–^ anion, and 9.5 water molecules of solvation. Essentially, the cation composition is the same as in **1**. The silver clusters in both compounds maintain Ag···Ag distances similar to those found in **1** (Table 2).

The number and coordination geometry of the Ag(I) ions in compound **2** are identical to those of the cations in **1**, with the exception of Ag8 (Figure S10). The average Ag–N distances remain unchanged compared to those in **1**, and the Ag–S and Ag–O bond lengths are slightly longer. In contrast to compound **1**, where the coordinated methanol molecule is present, the replacement of this molecule by water results in a shorter Ag5–O10 bond in compound **2**, in comparison to the Ag5–O50 bond observed in compound **1**. Furthermore, the nitrate ion attached to Ag5 undergoes a change in coordination mode, from a symmetric bidentate terminal in **1** to a bridging (bidentate, monodentate) nitrate in **2**. In this mode, O11 and O12 asymmetrically coordinate to Ag5, while O13 coordinates to the Ag8^b^ (b: x, y 1+z) of a neighboring cation (Figure S10).^55^ Consequently, cations are linked to one another, forming chains along the *c*-axis in a head-to-tail configuration (Figure S11). Now, with a τ_4_ value of 0.67, the coordination environment of Ag8 is best described as a distorted seesaw-shaped four-coordinate geometry.^51^

In **2**, weak contacts are observed between the S2 and S3 atoms of the thiazolidin-one rings of Type B ligands and the Ag8 and Ag5 ions, respectively. These contacts are slightly longer than those observed in **1** (Table 2). Furthermore, there are two classic intracation hydrogen bonds where the N25–H25A and N45–H45A bonds of the amine groups of two organic ligands act as donors, and the atoms O33 and O22 of two nitrate ligands serve as acceptors (Figure S11, Table S3). The remaining N–H bonds of the amine groups also engage in other intermolecular hydrogen bonds with the oxygen atoms of the crystallization water molecules and with some oxygen atoms of the nitrates (Figure S11).

**Figure 5:**
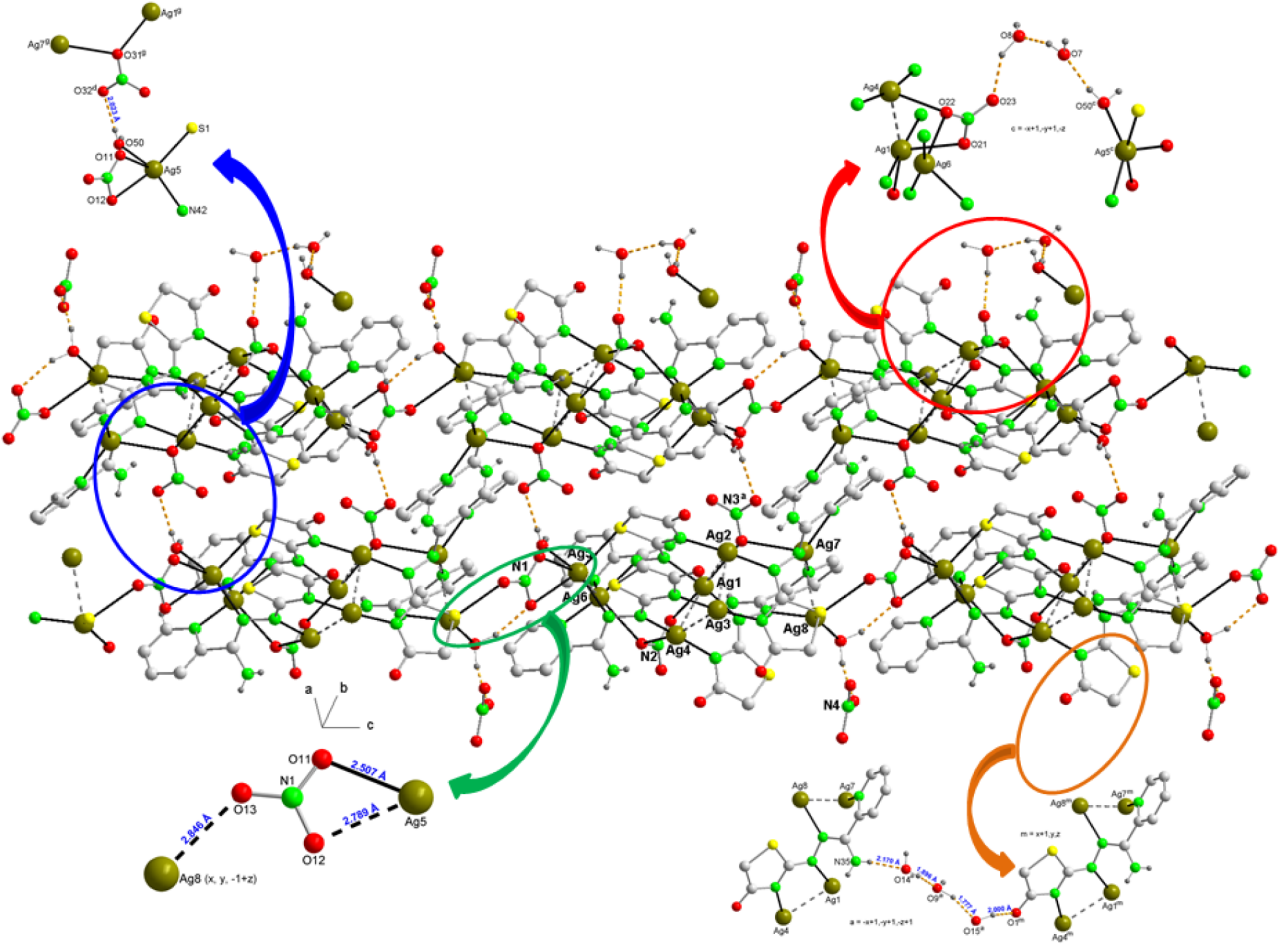
The crystal packing of compound 2. Several O–H···O hydrogen bonds are shown as dashed lines.

In the crystal packing of **2**, the hydrogen bond N25–H25B···O41^*e*^ (*e*: −*x* + 1, −*y*, −*z* + 1) is noteworthy, supporting each cation’s interaction with the corresponding nitrate anion (Figure 5). In addition, hydrogen bonds formed between the coordinated water molecules (O10 and O20) as hydrogen bond donors, and the O23 and O43 oxygen atoms of a coordinated nitrate (N2) and the nitrate anion (N4) as acceptors are of interest. These bonds connect polymers along the *b*-axis, with each nitrate anion acting as a bridge between the amine group (N25) of each cation and the coordinated water molecule (O20) of a neighboring cation (Figure 5) (Table S3).

In short, a two-dimensional network extending parallel to the bc-plane is formed in compound **2**. Additionally, although the hydrogen atoms of the crystallization water molecules could not be located, the distances between the oxygen atoms, as well as those with some of the nitrates averaging 2.777 Å, support the formation and stability of a 3D network. As expected, **2** also exhibits various intercation interactions, including ring···ring, silver···ring, and X–O···ring (where X = C or N), similar to those described in **1**. These interactions contribute to the overall structure and are further detailed in Figure S12 and Table S4.

#### 3.1.3 Structures of {[Ag_8_(AmDHotaz)_4_(NO_3_)_2_(H_2_O)_2_](NO_3_)(OH)·6H_2_O}_n_ (**3**) and {[Ag_8_(AmDHotaz)_4_(NO_3_)_2_(H_2_O)](NO_3_)(OH)·4.5H_2_O}_n_ (**3a**)

**Figure 6:**
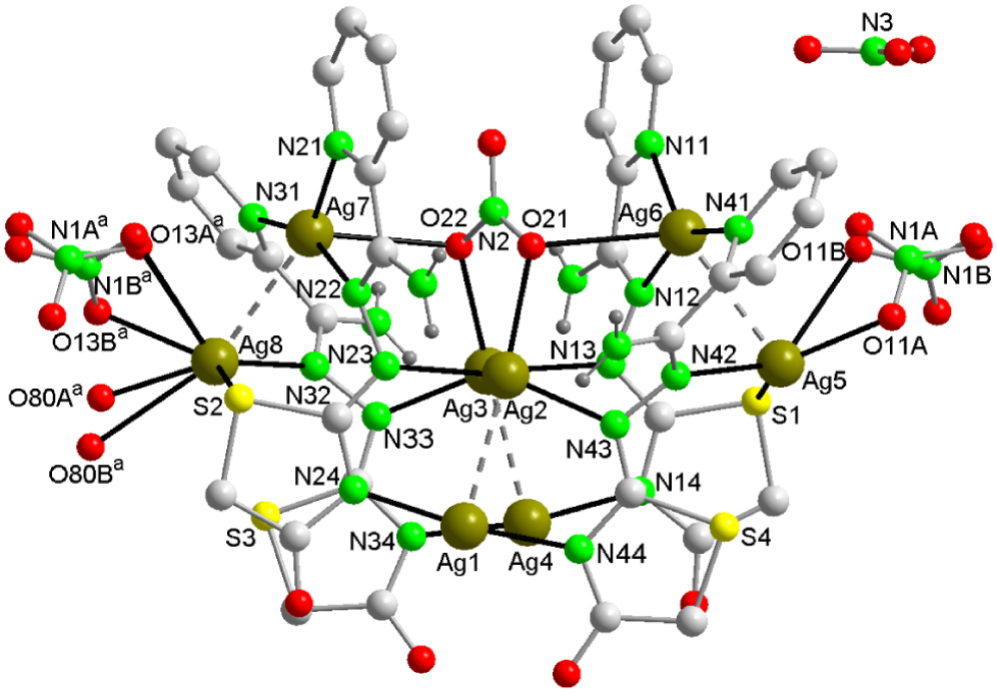
View of the asymmetric unit of **3a** where the Ag_4_ and Ag_2_ aggregates are interconnected through Ag–N, Ag–O, and Ag–S. Crystallization water molecules are omitted for clarity. Color code: Ag, dark yellow; S, yellow; C, pale gray; O, red; N, bright green, H, dark gray.

Both compounds are isostructural, differing only in the number of crystallization molecules. This compound crystallizes in the orthorhombic space group *Pna*2_1_, and its asymmetric unit comprises eight silver atoms, four AmDHotaz ligands, three nitrate ions, a hydroxide anion and 4.5 or 6 crystallization water molecules, forming a hydrated salt. In this salt, the cation is constituted by eight Ag atoms coordinated by four organic monoanions, two nitrate ions, and one water molecule (Figure 6). Two of the nitrate anions are ordered, but the third is disordered in two different orientations with occupancy factors of 0.43(4)/0.57(4). The cationic constitution is based on a central cluster of four Ag^+^ ions with three Ag–Ag distances ranging from 2.828(2) to 3.051(2) Å, comparable to the average Ag–Ag distance of 3.005 Å found in the CCDC (Table 2).

It has been postulated that the cation–anion charge equilibrium may be due to an OH^–^. This is based on the observation that the residual electron density data, which have been repeatedly reviewed and analyzed, have not found peaks that could be an indication of the presence of a fourth nitrate anion. To also rule out the existence of a carbonate instead of ionic nitrate, structures **3** and **3a** were reresolved by substituting ionic nitrate for a carbonate. The results obtained were worse than the previous ones. Furthermore, the IR spectra of both compounds were analyzed and no bands attributable to the presence of the carbonate ion were located. The new IR spectrum is now included as Figures S3b and S4b.

In contrast to the previous compounds, this compound exhibits a distinctive feature: the fourth Ag1–Ag4 distance of 3.425(2) Å is close to the sum of the van der Waals radii of two silver atoms (3.44 Å). This suggests an absence of interaction between these atoms, implying that the (Ag^+^)_4_ cluster does not form a cyclic shape but rather adopts a U-shaped structure. Along the “b” axis, at average distances of 5.26 and 5.22 Å in opposite directions, pairs of Ag^+^ ions (Ag5–Ag6 and Ag7–Ag8) are positioned, separated by distances of 2.971(2) and 2.921(2) Å, respectively. These distances are slightly longer than those observed in compounds **1** and **2**. The eight Ag^+^ ions are coordinated by the donor atoms of the four organic ligands and two nitrate ions. These nitrates act as bridging ligands with varying denticity, as previously described, resulting in coordination numbers and geometries that are not significantly different from those already detailed.

In the central Ag_4_ unit, the terminal ions Ag1 and Ag4 exhibit linear geometry and are each coordinated by the nitrogen atoms of two thiazol-4-one rings from different ligands, forming bond angles of 172.73° and 171.39°. The central ions, Ag2 and Ag3, display trigonal coordination from the hydrazine nitrogen atoms of two ligands, with angles of 154.23° for Ag2 and 159.73° for Ag3. The third coordination position is occupied by an oxygen atom from the nitrate N2, leading to a T-shaped coordination geometry.

The coordination in the two Ag_2_ dinuclear clusters flanking the central Ag_4_ cluster is similar to that observed in the structures of compounds **1** and **2**. In the Ag5–Ag6 cluster, Ag5 shows distorted planar trigonal coordination through AgNOS, involving the nitrogen atom N_az_ of ligand L4, the oxygen atom O11 of a nitrate, and the sulfur atom S1 of ligand L1. Meanwhile, Ag6 exhibits a seesaw coordination geometry through the nitrogen atoms N_py_ of ligands L1 and L4, the nitrogen atom N_az_ also from ligand L1, and the oxygen atom O21 from a second nitrate. In the Ag7–Ag8 cluster, Ag7 has the same coordination as Ag6, with the nitrogen atoms N_py_ of ligands L2 and L3, and the oxygen atom O22 of the same nitrate. Ag8, like Ag5, is coordinated by the sulfur atoms S2 and nitrogen N_az_ of ligands L2 and L3, respectively, and the oxygen atom O13 of a nitrate in the position *x*, −1 + *y*, *z*. Moreover, Ag8 coordinates with the oxygen atom O80 of a water molecule, adopting a distorted seesaw geometry.

In compound **3**, the Ag–N distances are comparable to those in the previously described structures (Table 2), averaging 2.275 Å for the distances involving the nitrogen atoms of the imino-pyridine groups (average Ag–N_py_, 2.263 Å; Ag–N_az_, 2.283 Å), and 2.142 Å for distances involving the nitrogen atoms of the imino-thiazolidine groups (average Ag–N_hy_, 2.191 Å; Ag–N_taz_, 2.091 Å).

Two of the ligands, L1 and L2, also coordinate to the outermost silver atoms of the cluster (Ag5 and Ag8) through the S1 and S2 sulfur atoms of the thiazolidin-4-one rings (Table 2). These distances are roughly equal to the average found in the CCDC Database. Furthermore, the sulfur atoms S3 and S4 of the other two ligands are at distances of 3.319(4) and 3.117(4) Å, respectively, from the aforementioned silver atoms. These distances are less than the sum of the van der Waals radii of Ag and S, suggesting the presence of some weak Ag···S interactions.

**Figure 7:**
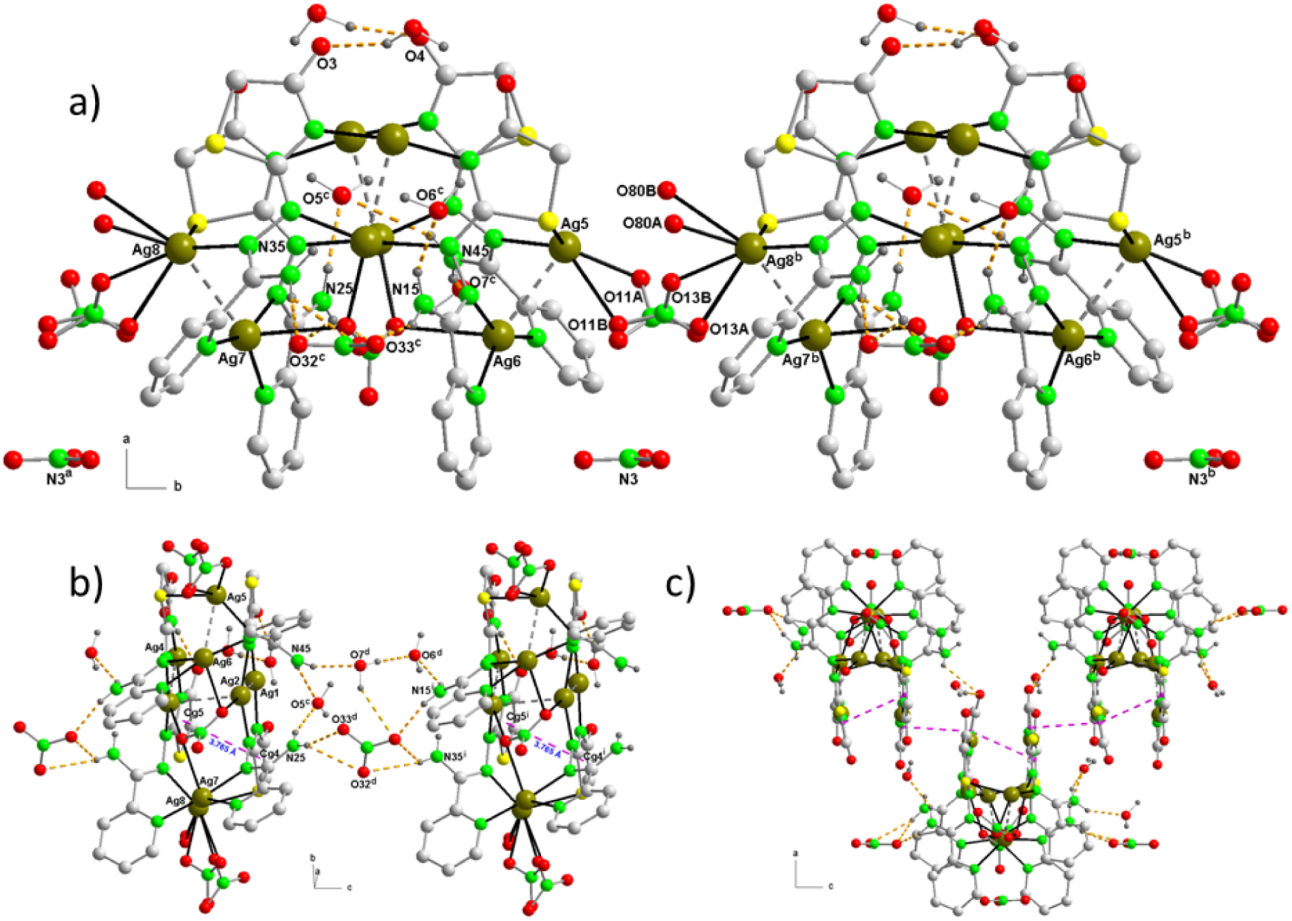
A partial packing diagram for **3a**, a) with chains running along the “b” axis showing a asymmetric tridentate bridge nitrate between nearest neighbor cations, b) one-dimensional supramolecular chain viewed down the “c” axis constructed by hydrogen-bonding patterns between the nitrate anion, the water molecules and the amine groups and c) 2D supramolecular architecture formed by hydrogen-bonding and π–π interactions along the “ac” plane. Hydrogen bonds are shown as orange dashed lines. The symmetry codes are as in Tables S5 and S6.

The oxygen atoms of the nitrate ligands in compound **3** act as bridges between metal ions (Figure S13). The nitrate represented by N2 coordinates the Ag2 and Ag3 atoms of the central Ag4 cluster and the Ag6 and Ag7 atoms of the outer dinuclear clusters within the cation itself, as a bidentate bridge, with an average Ag–O bond distance of 2.442(11) Å and, at somewhat greater distances, an average of 2.792(11) Å (Table 2). The other nitrate, represented by N1, is disordered over two positions with occupancies of 0.55 and 0.45 (Figure 7a). It coordinates to the Ag5 and Ag8^b^ (*b*: *x*, 1 + *y*, *z*) atoms of neighboring clusters along the “b” axis, using the three oxygen atoms and acting as a bidentate, monodentate, or asymmetric tridentate bridge. Two of the Ag–O distances in this mode are significantly higher than the average Ag–O distance of 2.446 Å found in the CCDC database (Table 2).

**Figure 8:**
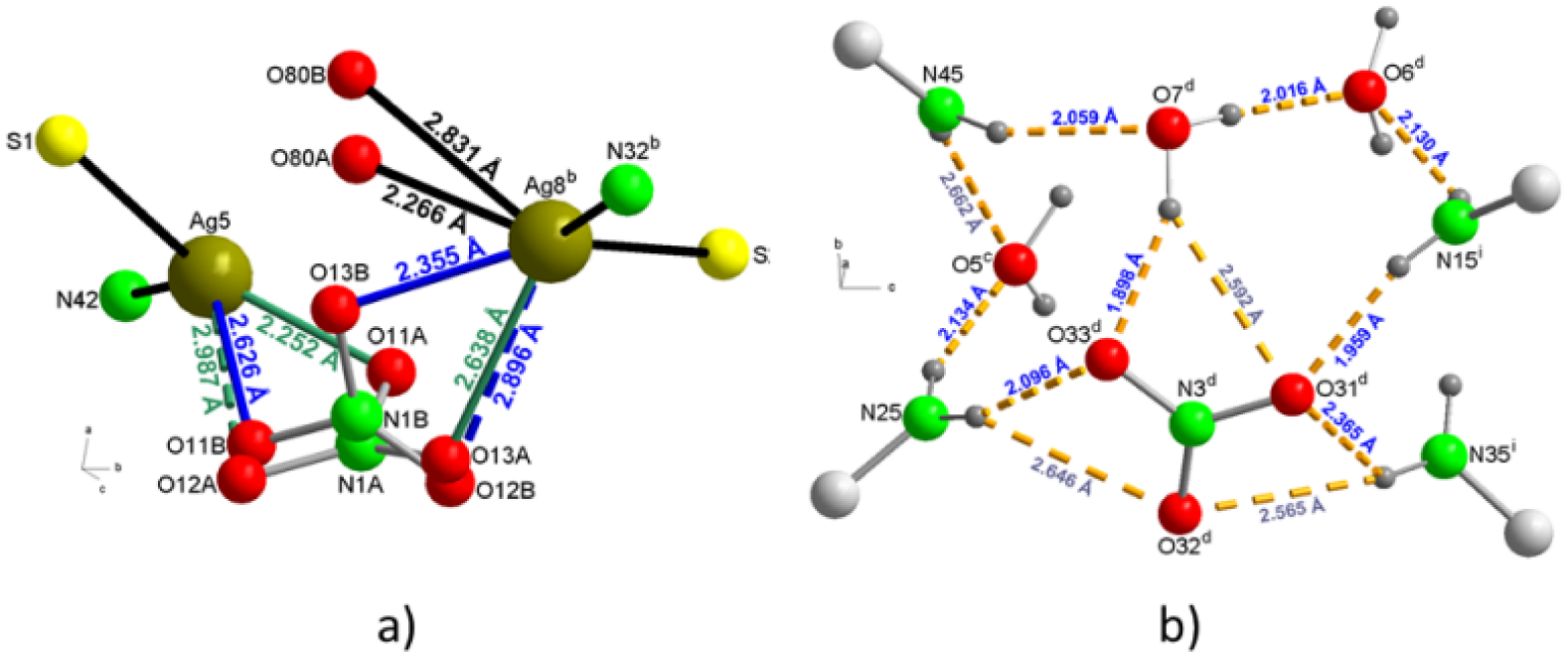
a) Representation of the nitrate bridge between two nearest neighboring cations in a chain, in the direction parallel to the b axis, in the packing of **3a** and b) highlight (4), (8), and (10) hydrogen-bonding patterns between the nitrate anion, the water molecules and the amine groups of four (AmDHotaz)^−^ ligands in two neighboring chains.

Additionally, the Ag8 atom is coordinated by a water molecule, O80, which is disordered over two positions with occupancy factors of 0.55/0.45. The respective distances are 2.27(2) Å for Ag8–O80A and 2.83(3) Å for Ag8–O80B. Consequently, the cations form chains in the “ab” plane along the crystallographic direction [010], which corresponds to the “b” axis, with an Ag5···Ag8^b^ separation of 5.075 Å (Figure 7a and 8a). The third nitrate, represented by N3, participates in forming interionic hydrogen bonds, with its oxygen atoms acting as acceptors and the N–H bonds of the amino groups of the ligands as donors (Table S5). These bonds act as bridges between cations, forming alternating chains along the “c” axis (Figure 7b). These bridges are further reinforced by new intermolecular hydrogen bonds involving water molecules represented by the oxygen atoms O5, O6, and O7^56,57^ (Figure 8b, Table S5).

**Figure 9:**
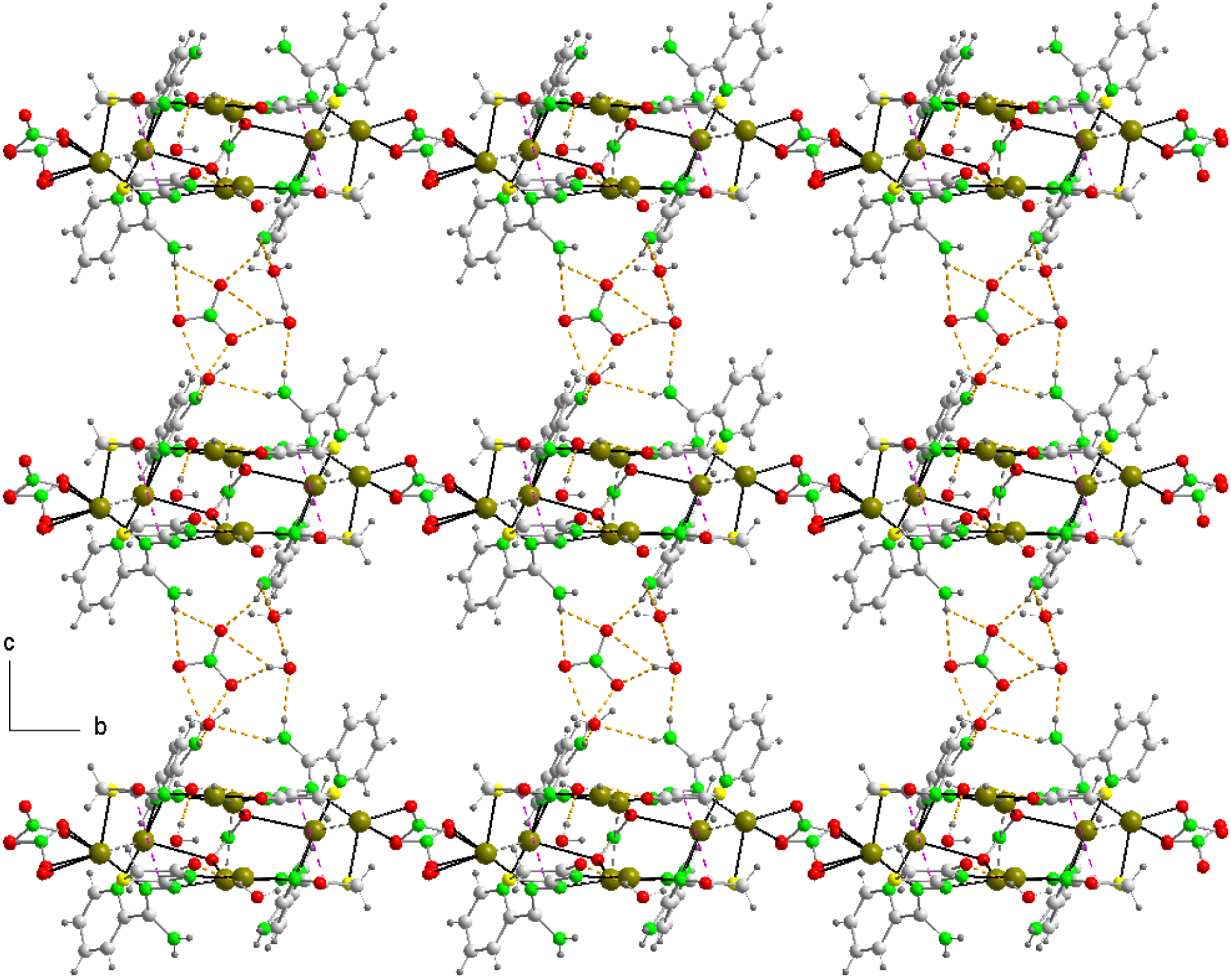
The crystal packing of compound 3a (along the a axis). Several N–H···O and O–H···O hydrogen bonds and π···π contacts that link the components in the crystal are shown as dashed lines.

In addition to these interactions, intramolecular π···π interactions occur between the thiazolidine rings of ligands L2 and L3, with a centroid distance of 3.765 Å (Table S6). There are also intermolecular stacking interactions between two neighboring cations, with ring centroid distances of the thiazolidine rings L3 and L4 at 3.713 Å. These rings stack in columnar formations along the crystallographic direction [001] (Figure 7c). As a result of these combined chains, double 2D sheets are formed parallel to the “bc” plane. These sheets feature holes with approximate dimensions of 13.54 × 4.11 Å^2^, which, when stacked to form a 3D network, create channels along the “a″ axis (Figure 9).

### 3.2 Theoretical Study

As mentioned in the previous section, the solid-state structure of all the reported octanuclear compounds is complex, characterized by the formation of various noncovalent interactions, predominantly hydrogen bonds and π-stacking interactions. These forces are well studied in general. Therefore, the DFT study focuses on other, less common aspects, such as Ag···S contacts. These contacts can be categorized as “elongated coordination bonds,” semicoordination bonds, or as examples of regium bonds (RgBs). The term RgB, referring to a noncovalent attractive contact between an electron donor and any group 11 atom acting as an electrophile, was coined by Stenlid and Brinck.^58,59^ It was initially used to explain interactions on the surfaces of metal nanoparticles. In this section, the argentophilic interactions and the ability of the heteroatoms of the ligand to act as Lewis bases are analyzed.

**Figure 10:**
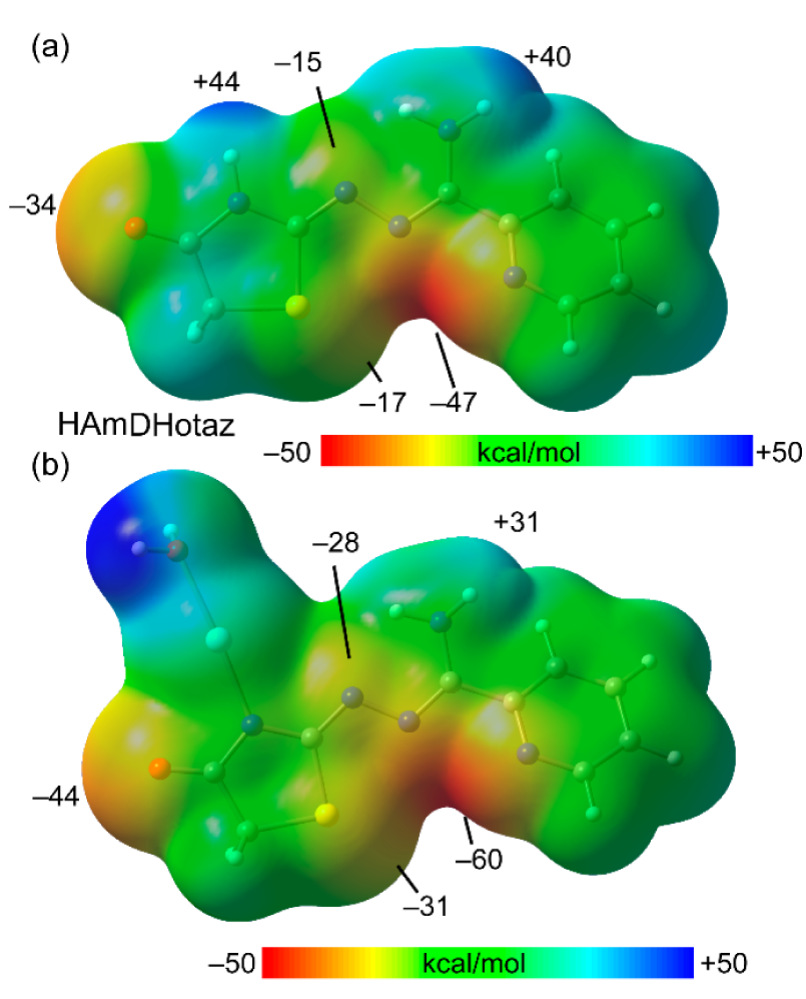
a) MEP surfaces of HAmDHotaz and b) its mononuclear complex with Ag(I). The MEP values at selected points of the surfaces are given in kcal/mol. Isovalue 0.001 a.u.

Figure 10 illustrates the molecular electrostatic potential (MEP) surfaces of the ligand and a model of the ligand where the most acidic H atom has been replaced by an Ag(I) ion. This substitution helps investigate how the coordinating atoms of the ligand are affected by metalation, aiding in rationalizing the binding modes observed by this polydentate ligand. The MEP of the free ligand (Figure 10a) indicates that the most acidic proton is the NH of the five-membered ring (MEP maximum), followed by the H atoms of the NH_2_ group. The MEP minimum is located near the N atoms of the pyridine and hydrazonamide moieties (−47 kcal/mol). The MEP at the other sp^2^ N atom of the hydrazonamide group is less negative (−15 kcal/mol) due to the influence of surrounding H atoms. Furthermore, the MEP values at the O and S atoms of the five-membered ring are large and negative (−34 kcal/mol and −17 kcal/mol, respectively). Upon deprotonation and coordination of Ag to the five-membered ring, the MEP values at potential coordination sites become more negative, enhancing their ability to coordinate with silver. Notably, the MEP values at the global minimum and at the S atom are significantly more negative (−60 and −31 kcal/mol, respectively) in the metalated compound. Overall, this MEP analysis helps explain the strong propensity of this ligand to form multiple coordination bonds with Ag(I) atoms.

**Figure 11:**
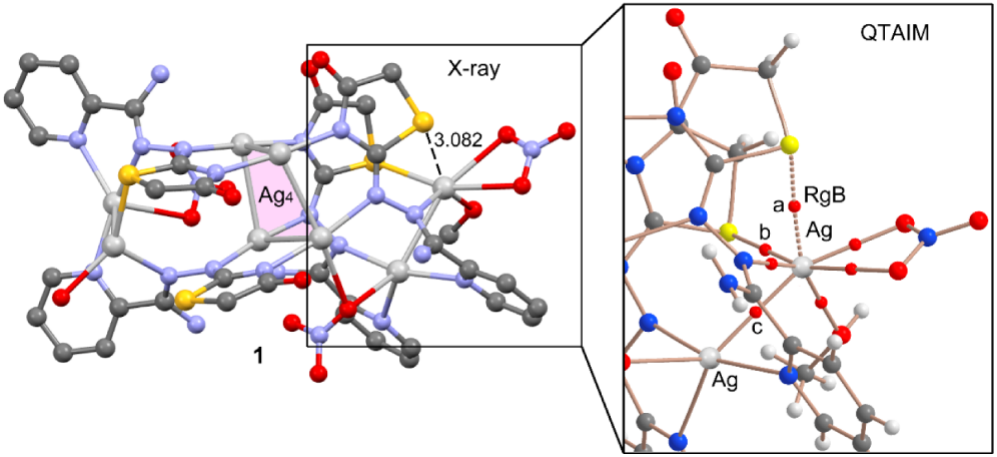
Left: Partial view of the X-ray structure of compound 1. H atoms omitted. Distance in Å. Right: Detail of the BCPs and bond paths (solid bonds) involving the Ag atom that participates in the RgB (marked as dashed bonds).

The study next examines the existence and nature of the long Ag···S contacts observed in the solid state of compounds **1**–**3**, as detailed in Figures 11, S14 and S15. In these compounds, besides forming a classical Ag–S coordination bond, the Ag ion also establishes a longer contact with the S atom of a nearby five-membered ring from another ligand. The distances in these contacts range from 3.082 Å in 1 to 3.113 Å in **3**. These distances are too long to be considered traditional coordination bonds but are shorter than the sum of the van der Waals radii of Ag and S (3.9 Å), suggesting they can be categorized as regium bonds (RgBs).

Quantum theory of atoms in molecule (QTAIM) analyses have been performed for all three compounds, and the results are included in Figures 11, S14, and S15. For clarity, only the bond critical points (BCPs) and bond paths starting from the Ag(I) ion involved in the RgB are represented. Each coordination bond is characterized by a BCP and bond path (depicted as solid lines) connecting the silver to the ligands (including EtOH and the anionic coligand). Notably, an additional bond CP and bond path connect the Ag-atom to the S atom located at 3.082 Å, represented as a dashed bond, confirming the existence of such interaction. To differentiate both Ag···S bonds (BCPs denoted as “a” and “b” in Figures 11, S14, and S15), the parameters at both BCPs are given in Table 3, along with the parameters of the Ag···Ag argentophilic interaction.

**Table 3:** Density (ρ), its Laplacian (∇^2^ρ), Potential Energy Density (*V*), Lagrankian Kinetic Energy Density (*G*), Total Energy density (*H*) in Atomic Units for the BCPs Labelled in Figures 11, S14 and S15

compound	BCP	ρ	∇^2^ρ	*V*	*G*	*H*	type
**1**	a	0.0191	0.0513	–0.0138	0.0133	–0.0005	RgB
	b	0.0439	0.1179	–0.0429	0.0362	–0.0067	Coord.
	c	0.0256	0.0668	–0.0235	0.0201	–0.0035	Ag···Ag
**2**	a	0.0182	0.0493	–0.0129	0.0126	–0.0003	RgB
	b	0.0403	0.1076	–0.0383	0.0326	–0.0057	Coord.
	c	0.0245	0.0634	–0.0220	0.0189	–0.0031	Ag···Ag
**3**	a	0.0177	0.0483	–0.0124	0.0122	–0.0002	RgB
	b	0.0516	0.1344	–0.0524	0.0430	–0.0094	Coord.
	c	0.0261	0.0660	–0.0236	0.0200	–0.0036	Ag···Ag

Literature sources^60−62^ suggest that interactions where the potential energy density (*V*) at the BCP is greater (in absolute value) than the Lagrangian kinetic energy (*G*) denote a covalent character (|*V*| > *G*). Noncovalent interactions are characterized by similar values of energy densities at the BCP (|*V*| ≈ *G*). Examining the values in Table 3 for all three complexes, it can be deduced that the Ag···S interaction is clearly noncovalent, with total energy densities (*H* = *V* + *G*) close to zero for the BCP denoted as “a.” Moreover, the electron density at this BCP is <0.02 au in all cases, typical for noncovalent bonding. In contrast, for the BCP denoted as “b,” the values of potential energy densities are greater (in absolute value) than the Lagrangian kinetic energy |*V*| > *G*, and the electron densities are ρ > 0.04 au, typical in coordination bonds. As for the argentophilic interactions, the densities are small (ρ < 0.04 au), and the *H* values are negative but smaller than those of the Ag–S coordination bond, indicating a partial covalent character.

**Figure 12:**
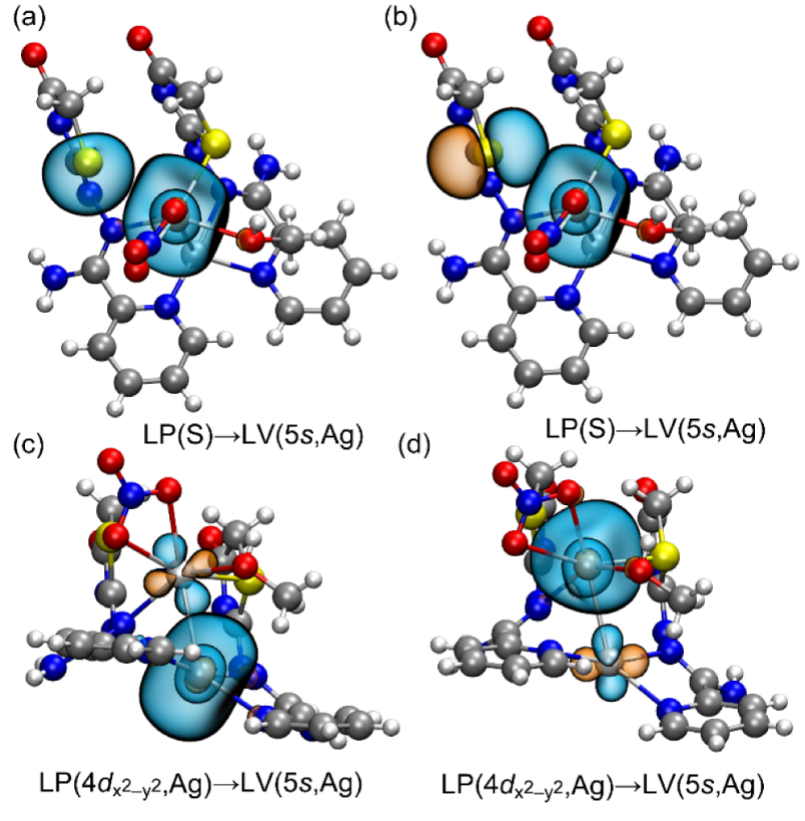
Representation of the NBOs involved in the electron transfer of compound 1 for the RgB (a,b) and Ag···Ag interactions (c,d).

We have further analyzed the Ag···S and Ag···Ag interactions from an orbital perspective, utilizing natural bond orbital (NBO) analysis. To facilitate this analysis, a dinuclear model of the complex, incorporating two silver atoms, two HAmDHotaz ligands, and coligands, was employed. Figure 12 illustrates the natural bond orbitals involved in the Ag···Ag argentophilic interactions and Ag···S interactions.

In the regium bonding interaction, there are two donor–acceptor interactions involving charge transfer from the lone pair (LP) located on the S-atom to the empty lone valence (LV) orbital, which consists of the 5s atomic orbital. For the argentophilic interaction, electron donation occurs from an LP of one silver (primarily composed of the 4d_*x*_2_–y_2 orbital) to the same LV orbital (5s) involved in the RgB of the other silver, and vice versa (Figure 12, bottom panel).

The total second-order stabilization energies (*E*^(2)^) due to the LP(S) → LV(Ag) and LP(Ag) → LV(Ag) donor–acceptor interactions are detailed in Table 4. These energies indicate that the stabilization contributions are greater for the argentophilic interactions than for the regium bonds. Compound **3** exhibits the weakest argentophilic interaction, which corresponds with its longer Ag···Ag distance. Regarding the RgB, the stabilization energies decrease from compound **1** to **3** and this trend aligns with the experimental distances, which increase from **1** to **3**, thereby reducing the orbital overlap.

**Table 4:** NBOs and Second Order Stabilization Energies for the Donor–Acceptor Interactions of Compounds **1**–3^a^

compound	donor	acceptor	*E*^(2)^
**1**	LP(S)	LV(S)	9.38
	LP(Ag)	LV(Ag)	11.48
**2**	LP(S)	LV(S)	8.41
	LP(Ag)	LV(Ag)	13.09
**3**	LP(S)	LV(S)	7.02
	LP(Ag)	LV(Ag)	8.85

a Energies in kcal/mol.

**Figure 13:**
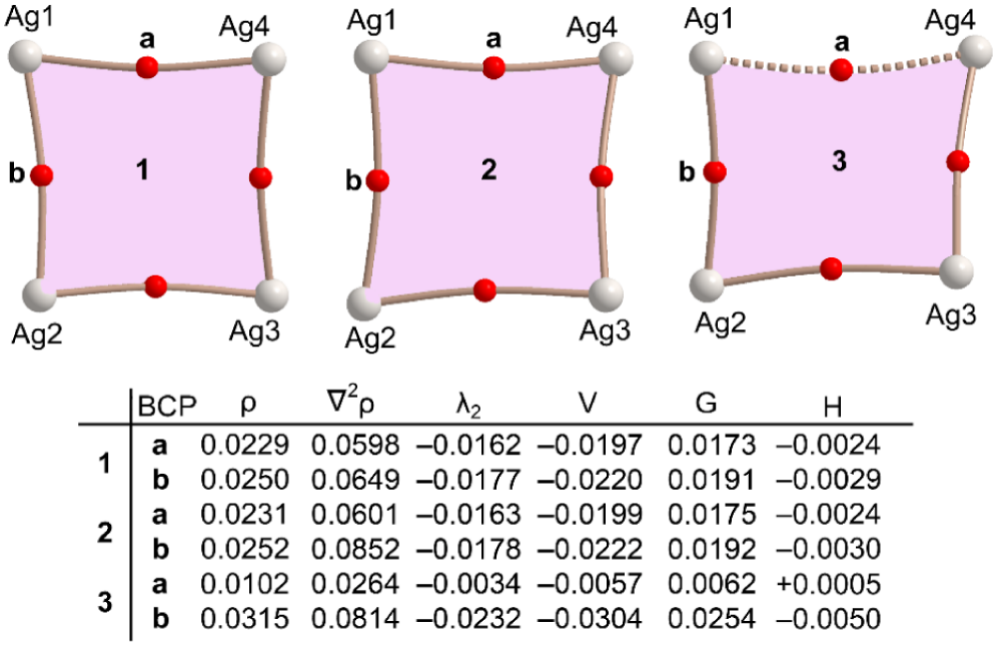
QTAIM analysis of the Ag4 clusters of compounds **1**–**3** and the values of density (ρ, a.u.), its Laplacian (∇^2^ρ), λ_2_ eigenvalues, potential energy density *V*, Lagrangian kinetic energy *G*(*r*), and total electron density energy values (*H*) in a.u. at the BCPs (red spheres) **a** and **b**.

**Figure 14:**
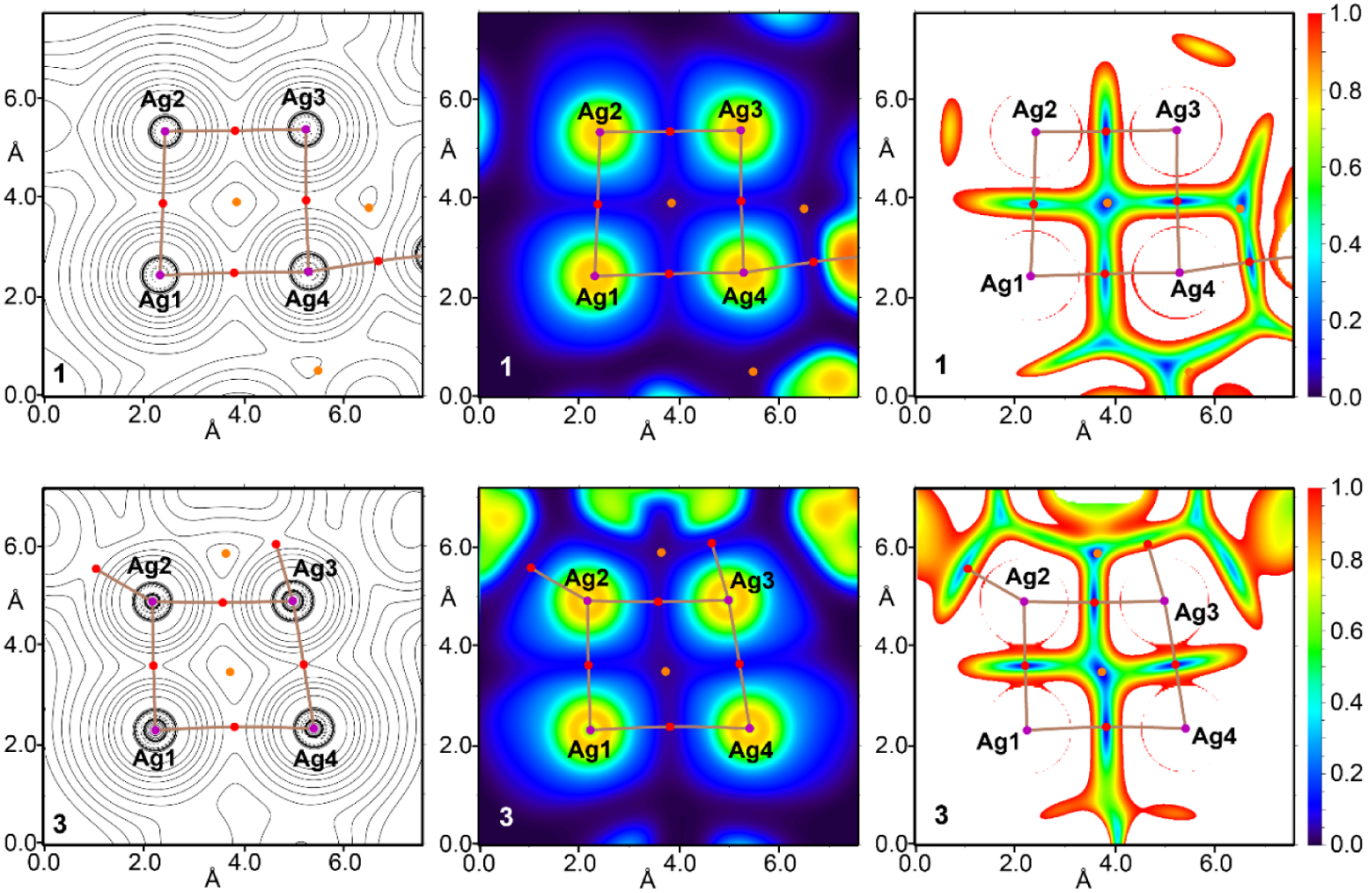
Contour line diagram of the Laplacian of electron density distribution ∇^2^ρ(*r*), bond paths, (left panel), visualization of electron localization function (ELF, center panel) and reduced density gradient (RDG, right panel) analyses for metallophilic interactions in the X-ray structures **1** (top) and **3** (bottom). Bond critical points (3, – 1) are shown in red, nuclear critical points (3, + 3) in purple and ring critical points (3, + 1) in orange, bond paths are shown as pale brown lines, and the color scale for the ELF and RDG maps is presented in a.u.

Finally, the Ag_4_ cluster in compound **3** has been analyzed using QTAIM analysis, with a specific focus on the observed differences, particularly where one of the Ag···Ag distances is longer than the other three. The results are depicted in Figures 13 and 14, focusing only the Ag_4_ cluster of each compound for simplicity. In all cases, bond critical points (BCPs) and bond paths interconnect the four Ag-atoms, confirming the existence of argentophilic interactions. This is true even in compound **3**, despite its long Ag···Ag distance (3.424 Å), where all four Ag-atoms are interconnected. The low magnitude of the electron density (0.010–0.031 au), positive values of the Laplacian of electron density (0.026–0.060 au), and negative energy density (from −0.0057 to −0.0304 au) in BCPs for short contacts Ag···Ag are typical for metallophilic interactions in similar chemical systems.^63,64^

The balance between the Lagrangian kinetic energy *G*(*r*) and potential energy density *V*(*r*) at the BCPs for metallophilic interactions in all BCPs apart from “a” in **3** reveals noticeable covalent contribution in all these short contacts.^65^ The Laplacian of electron density can be decomposed into the sum of contributions along the three principal axes of maximal variation, giving the three eigenvalues of the Hessian matrix (λ_1_, λ_2_, and λ_3_), and the sign of λ_2_ can be utilized to distinguish bonding (attractive, λ_2_*<* 0) weak interactions from nonbonding ones (repulsive, λ_2_*>* 0).^66^ Thus, the discussed metallophilic interactions Ag···Ag are attractive. The contour line diagrams of the Laplacian of electron density distribution ∇^2^ρ(*r*), bond paths, visualization of electron localization function (ELF) and reduced density gradient (RDG) analyses for metallophilic interactions Ag···Ag are shown in Figure 14. These 2D maps along the QTAIM parameters at the BCPs evidence that the argentophilic interactions in the Ag_4_ clusters of compounds **1** and **2** are akin to those in the isolated Ag_2_ moieties and previously reported clusters.^64^ However, in compound **3**, one of the Ag···Ag interactions exhibits a higher electron density at the BCP (BCP “b,” ρ = 0.0315 au), indicating increased electron sharing between the Ag ions. Consequently, due to the strengthening of the Ag1···Ag2 (and symmetrically equivalent Ag4···Ag3) interaction, the Ag1···Ag4 bond weakens (BCP “a,” ρ = 0.0102 au) and becomes clearly noncovalent, as indicated by the positive value of the total energy density (*H*) and the reduced ELF at the Ag1···Ag4 BCP (see Figure 14).

## 4 Conclusions

This article presents the synthesis and comprehensive characterization of five new silver octanuclear clusters (I), using IR spectroscopy, elemental analysis, and X-ray crystallography. The study reveals the presence of argentophilic interactions within these compounds, supported by crystallographic studies and DFT analysis. The multifunctional ligand HAmDHotaz, which is based on 4-oxothiazolidine and 2-pyridine rings linked via a hydrazine bridge, demonstrates unique coordination with Ag^+^ ions. In this context, the chelate hydrazine-pyridine unit plays a key role.

The reaction between HAmDHotaz and AgNO_3_ results in a series of octanuclear silver clusters, wherein the silver ions exhibit various coordination centers: 2 (Ag2, Ag3), 3 (Ag4, Ag8), and 4 (Ag1, Ag5, Ag6, Ag7). In these structures, four ligands act as bridges between different silver ions, providing stability, while NO_3_^–^ ions and some solvent molecules function as terminal ligands. The coexistence of solvates further impacts the supramolecular assembly, as evidenced by weak intermolecular interactions of the types N–H···O–N, O–H···O, N–H···O=C, C–H···O–N, and π–π stacking, along with argentophilic interactions. These interactions extend into 2D and 3D networks. Notably, the shortest Ag···Ag distances of 2.8277(15) and 2.8406(15) Å, found in {[Ag_8_(AmDHotaz)_4_(NO_3_)_2_(H_2_O)_2_](NO_3_)(OH)·6H_2_O}_*n*_ and {[Ag_8_(AmDHotaz)_4_(NO_3_)_2_(H_2_O)](NO_3_)(OH)·4.5H_2_O}_*n*_, exemplify unusually strong argentophilic interactions.

The compounds [Ag_8_(AmDHotaz)_4_(NO_3_)_3_(MeOH)(H_2_O)](NO_3_)·MeOH·7.5H_2_O and {[Ag_8_(AmDHotaz)_4_(NO_3_)_3_(H_2_O)_2_](NO_3_)·9.5(H_2_O)}_*n*_ are identified as solvomorphs. In both crystal structures, methanol and crystallization water molecules are retained through hydrogen bonding, contributing to the overall stability and structure.

Theoretical studies have shed light on the complex nature of argentophilic interactions within the Ag_4_ clusters. These analyses reveal that Ag···S contacts, longer than traditional coordination bonds, are characterized as regium bonds, indicative of unconventional bonding scenarios. Natural Bond Orbital (NBO) analyses underscore the importance of orbital interactions in stabilizing these cluster structures, while QTAIM analyses provide insights into the varying covalent characteristics of Ag···Ag interactions. Additionally, molecular electrostatic potential (MEP) studies demonstrate how ligand metalation impacts the coordination capabilities of the HAmDHotaz ligand, enhancing its ability to form multiple bonds with Ag(I) atoms.

The most recent advances in atomically precise silver nanoclusters protected by new type surface agents are based on the use of ligands that possess a multifunctional and polydentate coordination structure, constructed by nitrogen and occasionally sulfur donor atoms, such as the one described in the present work. These ligands contrast with different organic ligands such as thiolates, phosphines and alkynes, and inorganic ligands such as halides and chalcogenides, which have been used conventionally to date and have only one coordination position. Hopefully, in the near future, these new ligands will progress in protecting silver nanoclusters, as conventional ligands have done to date.

## Supporting Information Available

ESI–MS, FT-IR spectra, ^1^H and ^13^C NMR spectra, selected hydrogen bond lengths and angles, intermolecular ring–ring, ring-metal and CX-ring interaction parameters, and X-ray structure determination details (PDF)
