# Supplementary material for: Silver(I) Octanuclear Complexes Containing N′-(4-Oxotiazolidin-2-Iliden)picolinohydrazonamide and Nitrate as Bridge Ligands. An Example of Solvatomorphism?
Source: Inorg Chem. 2024 May 7;63(20):9221–36. doi: 10.1021/acs.inorgchem.4c00794 (PMC11110015; doi:10.1021/acs.inorgchem.4c00794)
Supplement: Supplementary file 1 — ic4c00794_si_001.pdf [file ic4c00794_si_001.pdf]

Electronic Supporting Information (ESI)

Silver(I) Octanuclear Complexes containing N'-(4-Oxotiazolidin-2-iliden)picolinohydrazonamide and nitrate as bridge ligands. An example of Solvatomorphism?

Isabel García-Santos[1]*, *Julia Krümpelmann*[1], *Manuel Saa*[1], *Sergi Burguera*[2], *Antonio Frontera*[2*], *Alfonso Castiñeiras*[1]*

[1] Department of Inorganic Chemistry, Faculty of Pharmacy, University of Santiago de Compostela, 15782 Santiago de Compostela, Spain

[2] Department de Química, Universitat de les Illes Balears, Crta. de Valldemossa km 7.5, 07122 Palma de Mallorca, Spain

alfonso.castineiras@usc.es (A. Castiñeiras); toni.frontera@uib.es (A. Frontera); isabel.garcia@usc.es (I. García-Santos)

Crystal Structure Determination S3

Figure S1. ESI-TOF mass spectrum of **1** S6

Figure S2. MALDI-TOF mass spectrum of **1**. S7

Figure S3. FT-IR spectra of **1** and **3** (4000-400 cm^{-1}). S8

Figure S4. FT-IR spectra of **1** and **3** (500-100 cm^{-1}). S10

Figure S5. ^{1}H NMR spectrum of **1** (300 MHz, DMSO-d_6). S12

Figure S6. ^{13}C NMR spectrum of **1** (300 MHz, DMSO-d_6). S13

Figure S7. a) A view of the supramolecular packing formed in **1** via N–H···O and O–H···O interactions, with dashed lines representing hydrogen bonds. The symmetry codes are as in Table S1. b) A view of π–π stacking interaction in **1**. Cg4 is the centroid of the thiazolidine ring 1, Cg7 is the centroid of the thiazolidine ring 4, Cg8 is the centroid of the pyridine ring 1 and C11 is the centroid of the pyridine ring 4 at (-x, 1-y, 1-z). S14

Figure S8. a**)** A view of silver(I)–aromatic ring and aromatic ring–aromatic ring intermolecular interactions at **1** and b) Hydrogen bond between cation and nitrate anions. The symmetry codes are as in Table S2 S15.

Figure S9. The crystal packing of compound **1** (along c axis). Several N–H···O and O–H···O hydrogen bonds and π···π contacts that link the components in the crystal are shown as dashed lines. S16

Figure S10. The cluster cation showing the coordination number and geometry in **2.** S17

Figure S11. A view of the supramolecular packing formed in **2** via N–H···O and O–H···O interactions, with orange dashed lines representing hydrogen bonds. The symmetry codes are as in Table S3. S17

Figure S12. Representation of inter- and intramolecular interactions for **2.** The symmetry codes are as in Table S4. S18

Figure S13. View of the cluster cation showing the coordination number and geometry at **3a**. S19

Figure S14. Partial view of the X-ray structure of **2** and detail of the BCPs and bond paths (solid bonds) involving the Ag atom that participates in the RgB S20.

Figure S15. Partial view of the X-ray structure of **3** and detail of the BCPs and bond paths (solid bonds) involving the Ag atom that participates in the RgB S20

Table S1. Hydrogen bond parameters for **1** S21.

Table S2. Intermolecular π–π, π–Ag and CX–π interactions in **1**. S21

Table S3. Hydrogen bond parameters for **2**. S22

Table S4. Intermolecular π–π, π–Ag and CX–π interactions in **2**. S22

Table S5. Hydrogen bond parameters for **3**. S23

Table S6. Intermolecular π–π, π–Ag and CX–π interactions in **3**. S23

Crystal Structure Determination:

A Colourless prismatic crystal of **$[Ag_8(DHotaz)_4(NO_3)_3(MeOH)(H_2O)](NO_3)\cdot MeOH\cdot 7,5H_2O$ (1)** was mounted on a glass fiber and used for data collection. Positional and anisotropic atomic displacement parameters were refined for all nonhydrogen atoms. Atoms in a NO_3^- ion (N4/O41/O42/O43) and in three water molecules (O10, O14 and O15) which showed very high thermal motion refined isotropically. Final refinement included the displacement-coefficient restrains ISOR of 0.001 for N3, N23, C15, C17 and C28 to prevent these atoms can be split into two positions. Hydrogen atoms bonded to carbon were placed geometrically and the O-H and N-H hydrogen atoms were initially positioned at sited determined from difference maps, but the positional parameters of all H atoms were included as fixed contributions riding on attached atoms with isotropic thermal parameters 1.2/1.5 times those of their carrier atoms. Nevertheless, the final results can be considered satisfactory from the chemical point of view.

A Colourless plate crystal of **$\{[Ag_8(DHotaz)_4(NO_3)_3(H_2O)_2](NO_3)\cdot 9,5(H_2O)\}_n$ (2)** was mounted on a glass fiber and used for data collection. Positional and anisotropic atomic displacement parameters were refined for most nonhydrogen atoms. But final refinement included the displacement-coefficient restrains ISOR of 0.001 for O14, O16, O17 and of 0.008 for O43 to prevent these atoms becoming 'non-positive definite' file and tables. Hydrogen atoms were located in difference maps and included as fixed contributions riding on attached atoms with isotropic thermal parameters 1.2/1.5 times those of their carrier atoms. The nitrate atoms (N4) and oxygen atoms of some crystallization water molecules (O14, O15, O16, O17) are affected with a considerable disorder degree as shown by its high anisotropic and thermal displacement parameters. It was not possible to localize this atom better. The final results can be considered satisfactory from the chemical point of view.

A Yellow prismatic crystal of **$\{[Ag_8(H_2O)_2(DHotaz)_4(NO_3)_3](NO_3)\cdot 11,5(H_2O)\}_n$ (2a)** was mounted on a glass fiber and used for data collection. Positional and anisotropic atomic displacement parameters were refined for most nonhydrogen atoms. But final refinement included the displacement-coefficient restrains ISOR of 0.001 for O3, N3, N12, N13, N33, N42, C17, C21, C27, C32, C33, C39, C45, C49 to prevent these atoms becoming 'non-positive definite', and atoms of the disordered nitrate anion N4 were refined isotropically. The crystallization water molecules, O17, O18, O19, O30 and O34 were also refined isotropically and their H atoms were not located. This has not been taken into account in the formula reported in the CIF file and tables. Two orientations observed of a disordered water of solvation (O17); site-occupation factors for the two orientations refined but constraines to sum to 1.0. Hydrogen atoms were located in difference maps and included as fixed contributions riding on attached atoms with isotropic thermal parameters 1.2/1.5 times those of their carrier atoms. Therefore, the contribution of the density of the disordered solvent molecules was subtracted from the measured structure factors with use of the SQUEEZE option [5]. Subsequent refinement then converged with R factors and parameter errors

significantly better than for all attempts to model the solvent disorder. The final results can be considered satisfactory from the chemical point of view.

A Colourless prismatic crystal of **$\{[Ag_8(DHotaz)_4(NO_3)_2(H_2O)_2](NO_3)(OH)\cdot 6H_2O\}_n$ (3)** was mounted on a glass fiber and used for data collection. Positional and anisotropic atomic displacement parameters were refined for most nonhydrogen atoms. But final refinement included the displacement-coefficient restrains ISOR of 0.001 for N1, O11, N33, C11, C26, C37 to prevent these atoms becoming 'non-positive definite', and atoms of the disordered water molecules (O50 and O80) were refined isotropically. A disorder of the water molecule O16 was refined isotropically on two positions with occupancies of 0.50/0.50. The crystallization water molecules, O9, and O15 were also refined isotropically and for O15 and O16 their H atoms were not located. This has not been taken into account in the formula reported in the CIF file and tables. Hydrogen atoms were initially positioned at sited determined from difference maps, but the positional parameters of all H atoms were included as fixed contributions riding on attached atoms with isotropic thermal parameters 1.2/1.5 times those of their carrier atoms. The structure was refined as a 2-component inversion twin with components 0.52(6), 0.48(6). The final results can be considered satisfactory from the chemical point of view.

A Colourless prismatic crystal of **$\{[Ag_8(DHotaz)_4(NO_3)_2(H_2O)](NO_3)(OH)\cdot 4{,}5H_2O\}_n$ (3a)** was mounted on a glass fiber and used for data collection. The final model has three NO_3^- for each complex with eight silver atoms. Two are ordered and the third is in two different orientations [occupancy factors 0.43(4)/0.57(4)]. I don't know if the electrical charges are balanced, but if there is an additional NO_3^- it has to be completely disordered, since you don't see anything resembling a nitrate in the residual electron density maps. Several H_2O's have been modeled, some ordered and some partially occupied. In some has been able to deduce and model the positions of the H based on the network of hydrogen bonds, but in others it is not possible. Positional and anisotropic atomic displacement parameters were refined for most nonhydrogen atoms. But final refinement included the displacement-coefficient restrains ISOR of 0.001 for C11, C26, C27 to prevent these atoms becoming 'non-positive definite', and atoms of the disordered nitrate and O80 were refined isotropically. A disorder of the water molecule O10 was refined isotropically on two positions with occupancies of 0.55/0.45. The crystallization water molecules, O8, and O9 were also refined anisotropically but their H atoms were not located. This has not been taken into account in the formula reported in the CIF file and tables. Hydrogen atoms were initially positioned at sited determined from difference maps, but the positional parameters of all H atoms were included as fixed contributions riding on attached atoms with isotropic thermal parameters 1.2/1.5 times those of their carrier atoms. The structure was refined as a 2-component inversion twin with components 0.49(6) and 0.51(6) [5]. The remains of electron density that remain are difficult to interpret. Some are even in absurd positions, such as at direct bonding distances from the ligand molecules. There is a small component of twinning that is seen in the diffraction images. Small enough that you can't process the data as a twin, but strong enough that it generates these meaningless

residual electron density remnants in the structure. The final results can be considered satisfactory from the chemical point of view.

RIAIDT
UNIDADE DE ESPECTROMETRIA DE MASAS
Edificio CACTUS * Campus Universitario Sur
15782 Santiago de Compostela (Spain)
Tel. 981 563 100. Ext. 16242 * Fax 981 547 077
E-mail: sxemasas@usc.es
Web: http://www.ti.usc.es/riaidt/masas/masas.htm

ESPECTRO DE MASAS ESI-TOF

Analysis Info

Analysis Name		Acquisition Date	
Method	cactus_positivo_alto_700-1800.m	Operator	Administrator
Sample Name		Instrument	micrOTOF

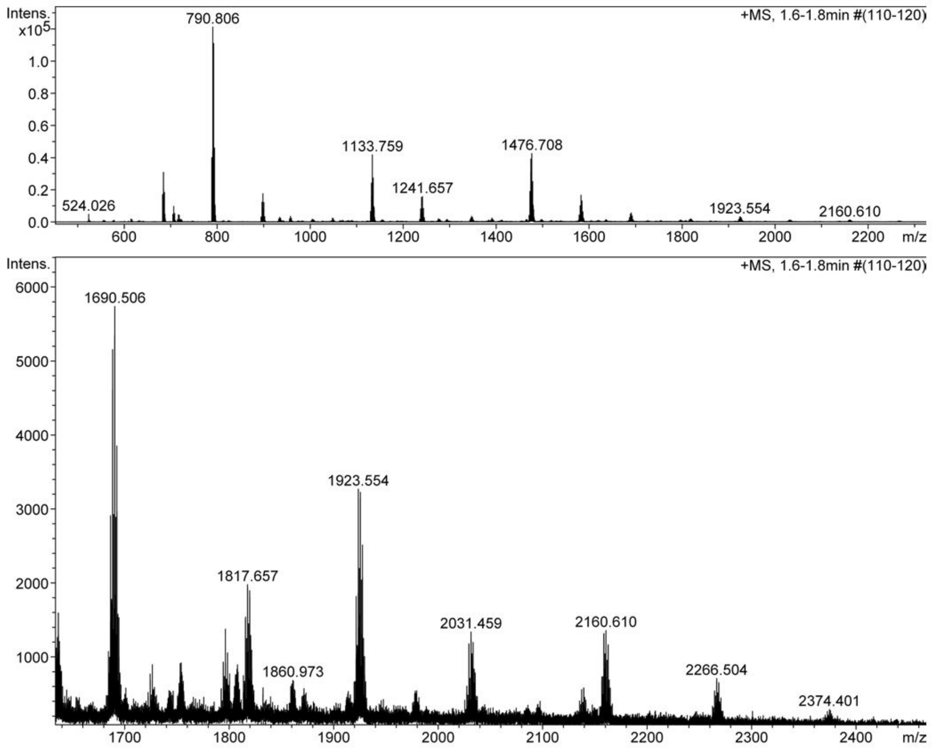

Bruker Daltonics DataAnalysis 3.3

Figure S1. ESI-TOF mass spectrum of compound **1**.

RIAIDT
UNIDADE DE ESPECTROMETRIA DE MASAS
Edificio CACTUS * Campus Universitario Sur
15782 Santiago de Compostela (Spain)
Tel. 981 563 100. Ext 16242 * Fax 981 547 077
E-mail: sxemasas@usc.es
Web: http://www.ti.usc.es/riaidt/masas/masas.htm

MALDI-TOF

Acquisition Parameter

Date of acquisition

Acquisition method name

D:\Methods\flexControlMethods\RP_1-3kDa_APR.par

Analysis Name

Instrument AUTOFLEX

Acquisition operation mode Reflector

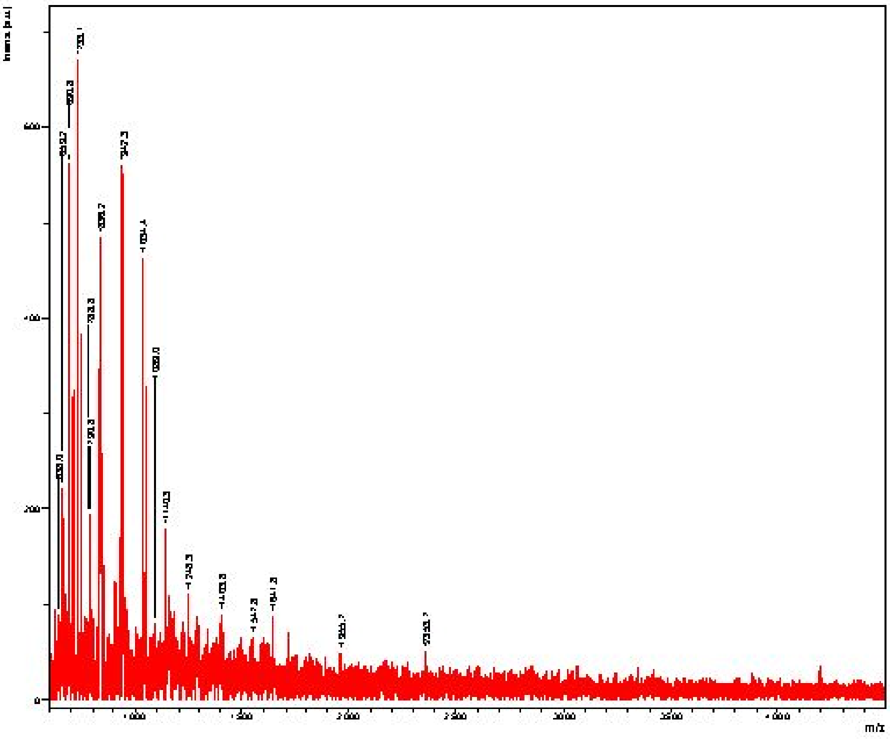

Bruker Daltonics flexAnalysis

Figure S2. MALDI-TOF mass spectrum of compound 1.

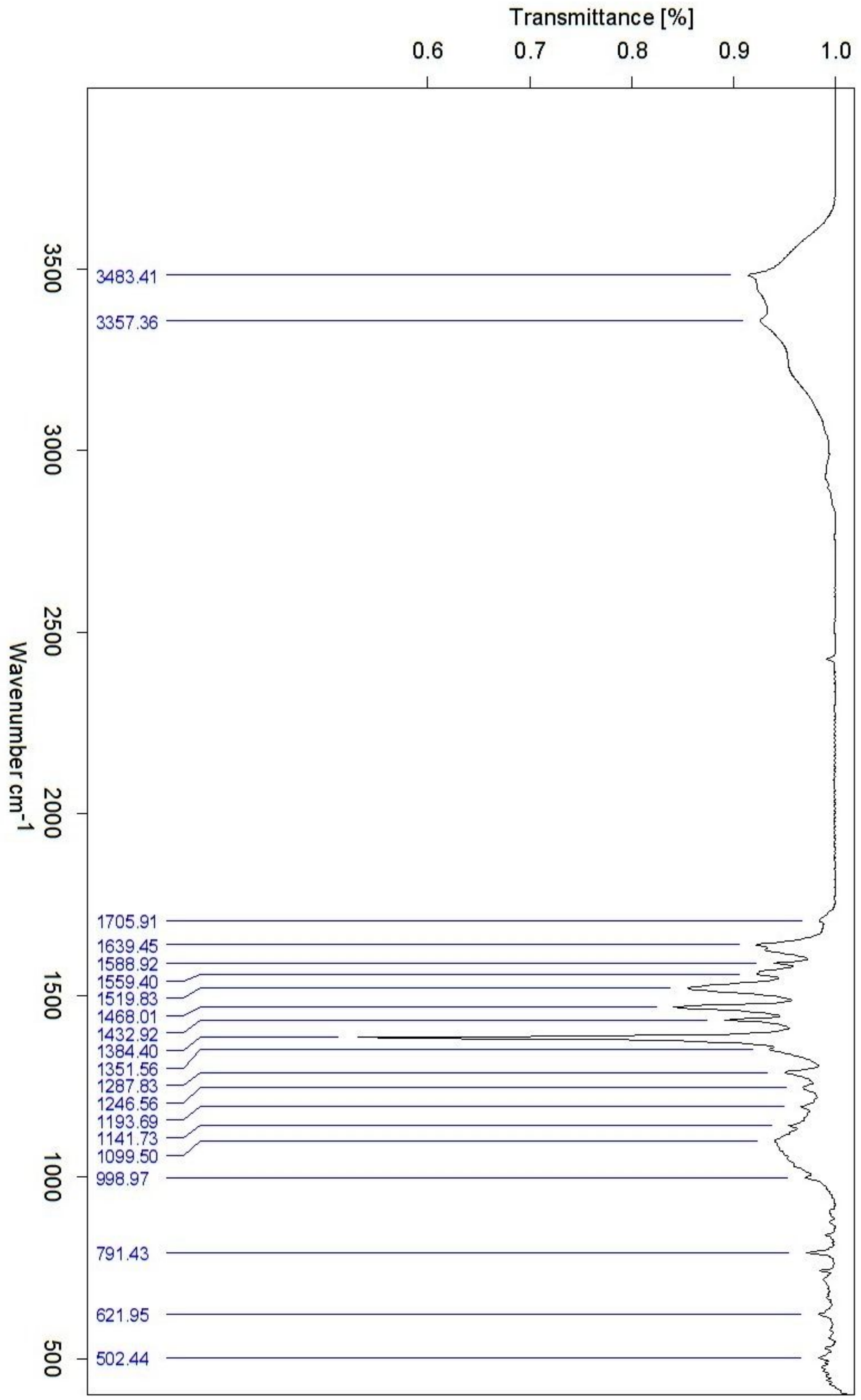

Figure S3a. FT-IR spectrum of compound **1** in the interval of 4000-400 cm^{-1}.

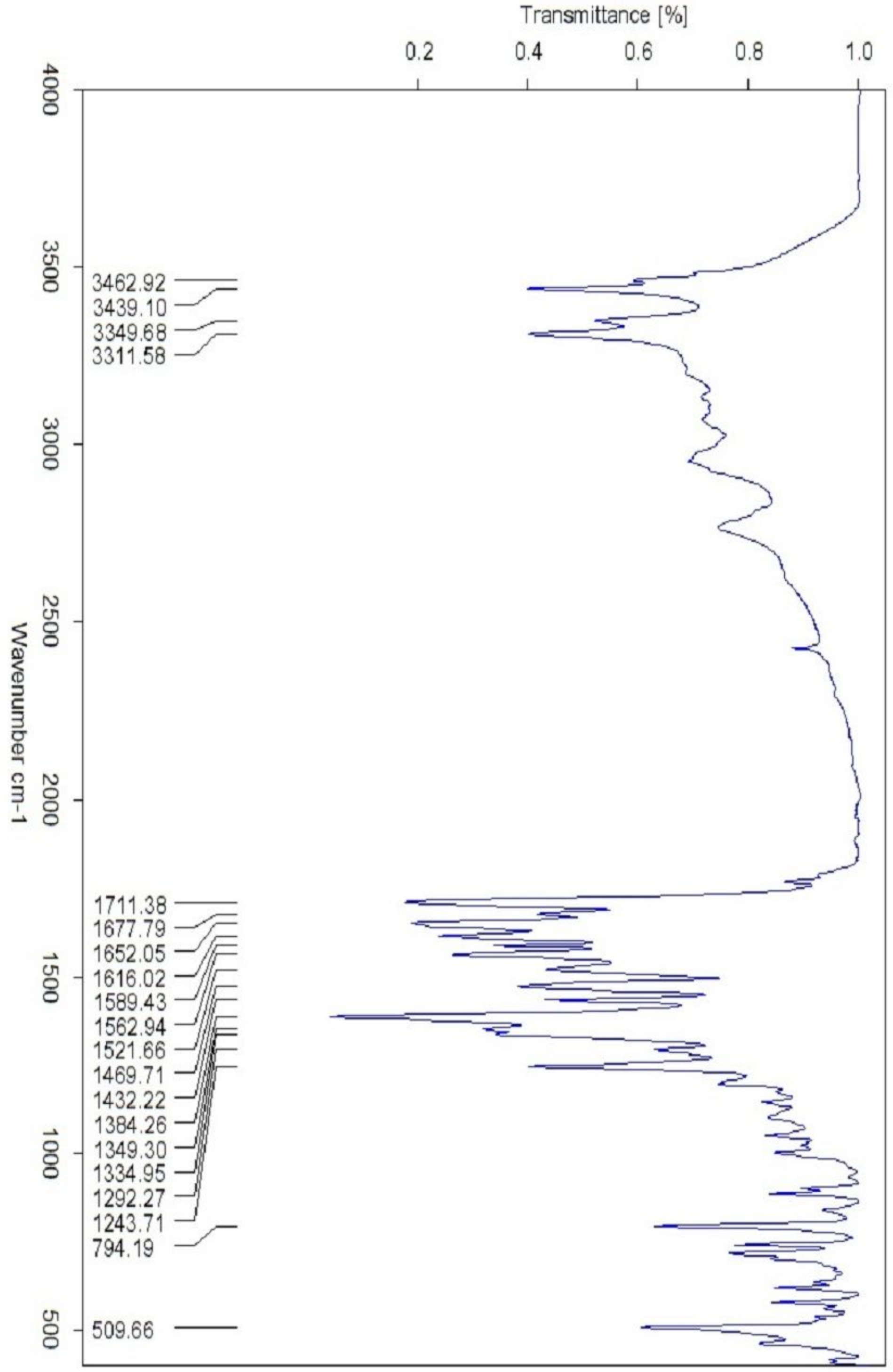

Figure S3b. FT-IR spectrum of compound **3** in the interval of 4000-400 cm^{-1}.

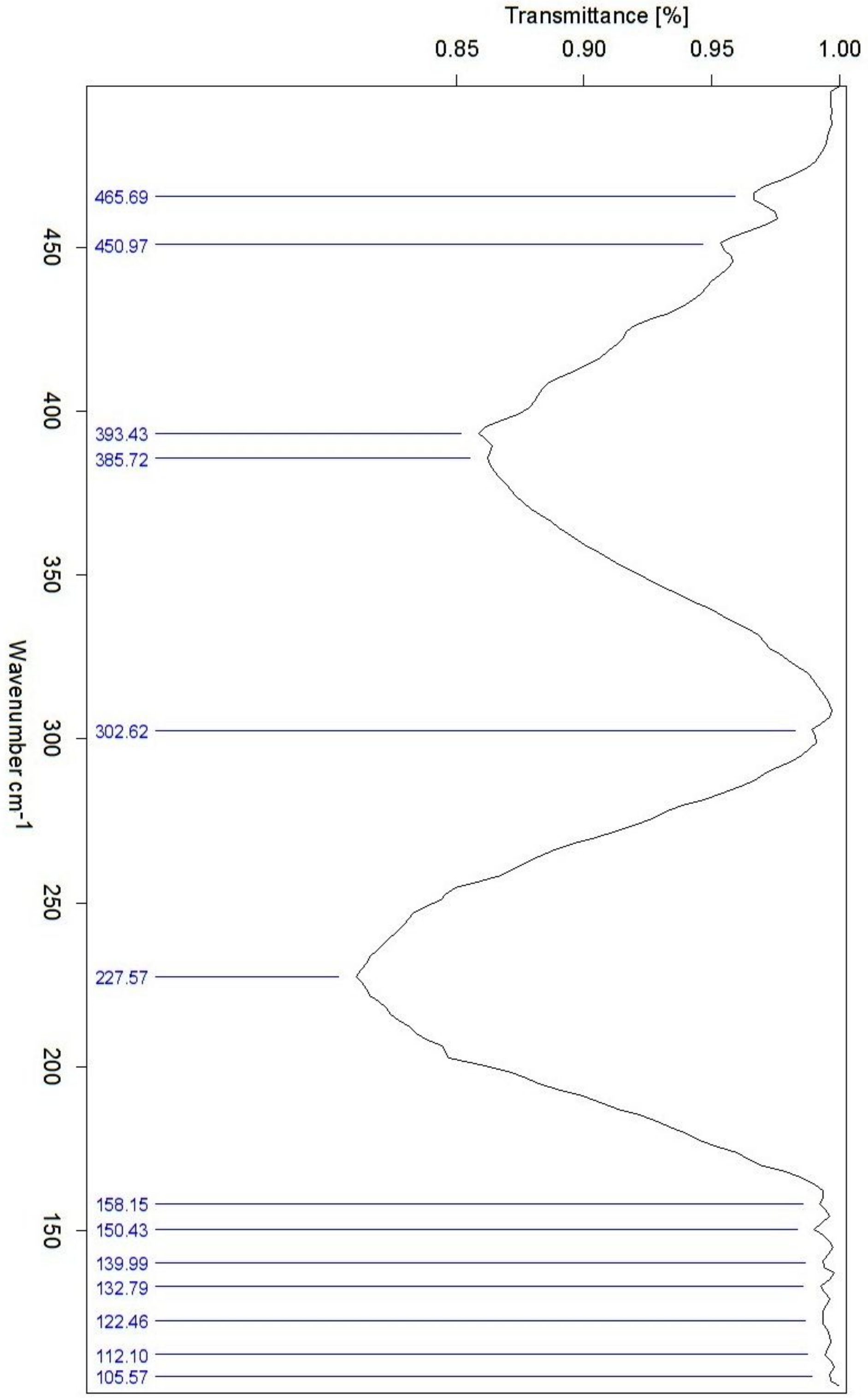

Figure S4a. FT-IR spectrum of compound **1** in the interval of 500-100 cm^{-1}.

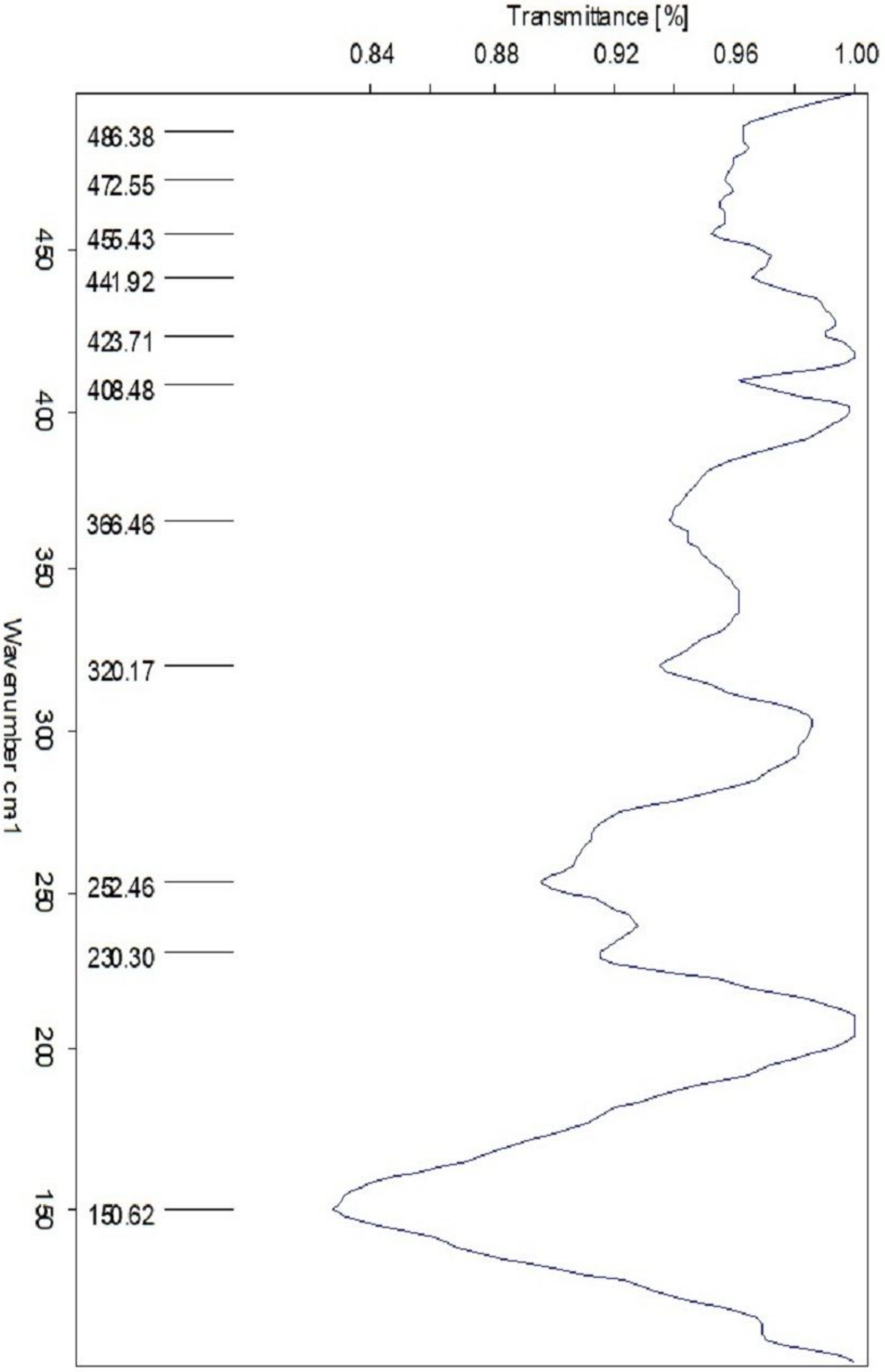

Figure S4b. FT-IR spectrum of compound **3** in the interval of 500-100 cm^{-1}.

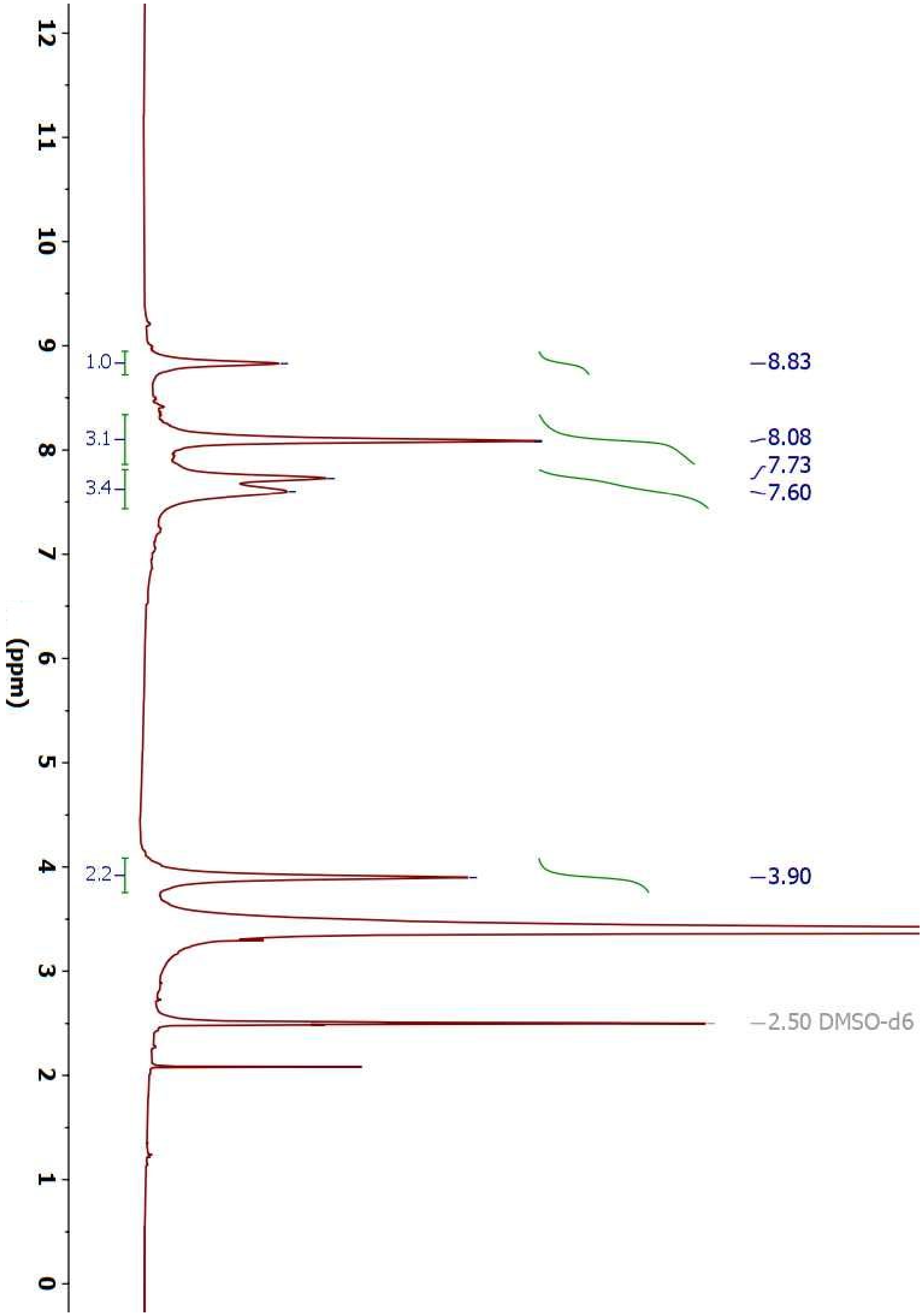

Figure S5. ^{1}H NMR spectrum of compound **1** (300 MHz, DMSO-d_6).

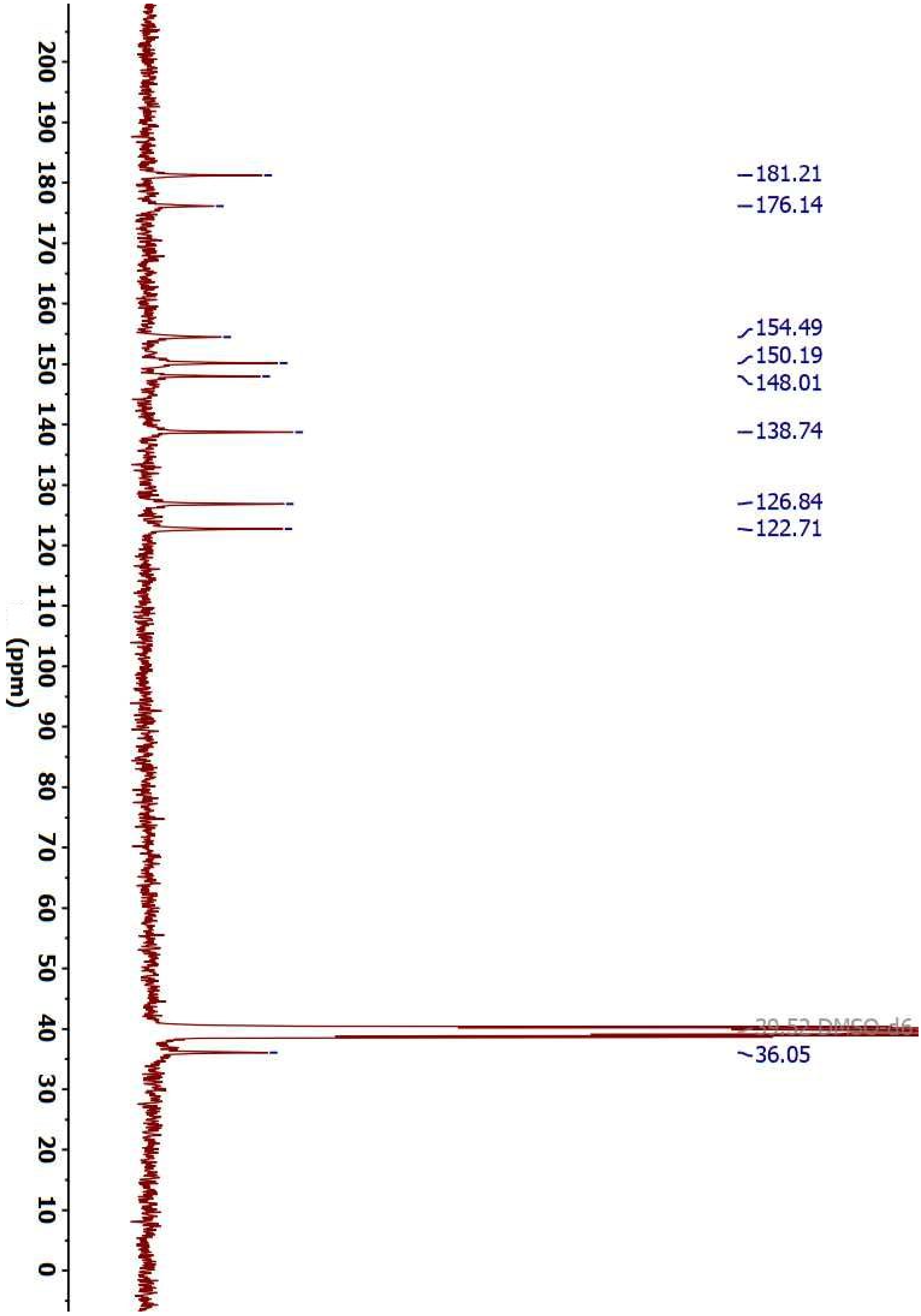

Figure S6. ^{13}C NMR spectrum of compound **1** (300 MHz, DMSO-d_6).

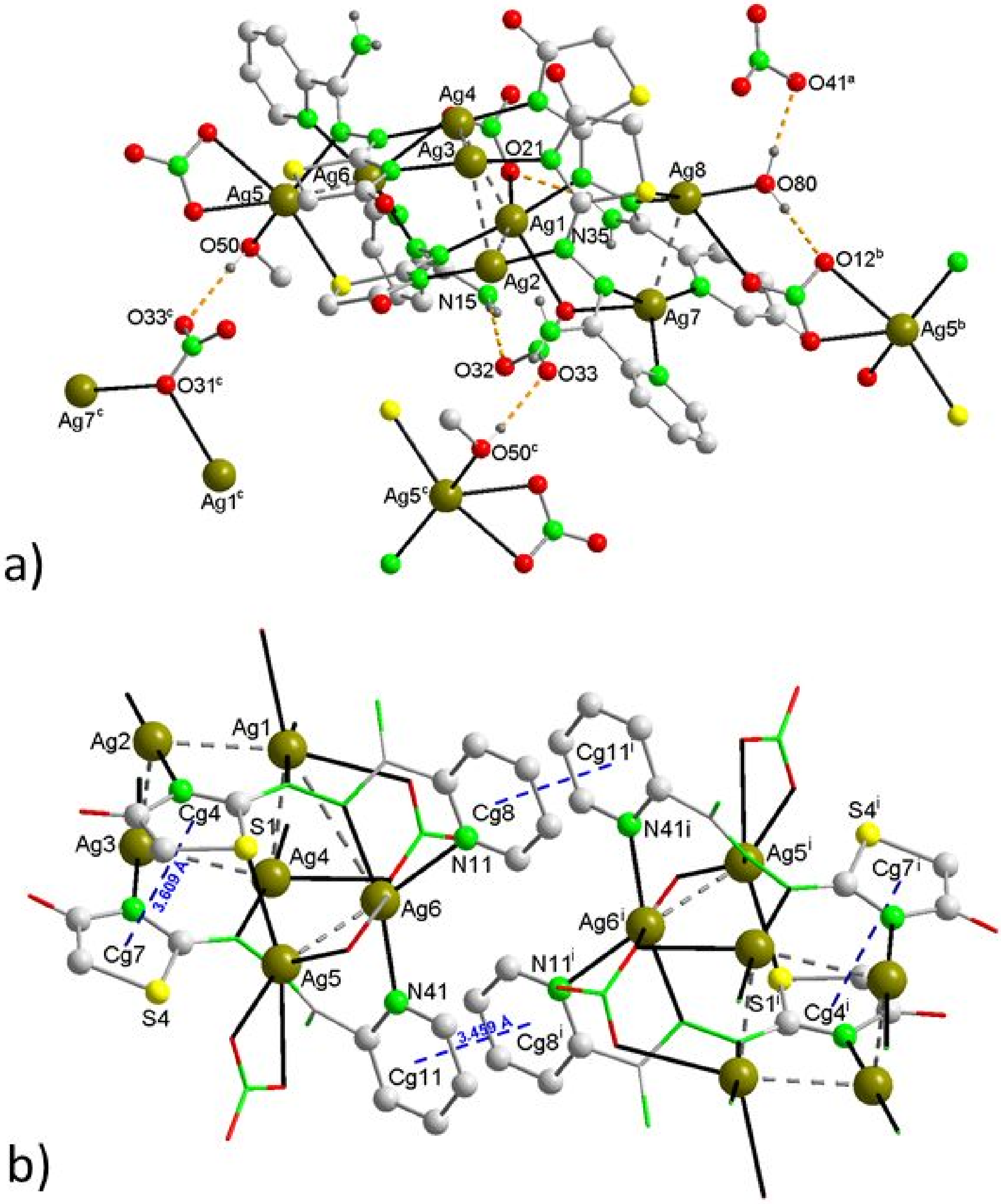

Figure S7. a) A view of the supramolecular packing formed in **1** via N–H···O and O–H···O interactions, with dashed lines representing hydrogen bonds. The symmetry codes are as in Table S1. b) A view of π–π stacking interaction in **1**. Cg4 is the centroid of the thiazolidine ring 1, Cg7 is the centroid of the thiazolidine ring 4, Cg8 is the centroid of the pyridine ring 1 and C11 is the centroid of the pyridine ring 4 at (-x, 1-y, 1-z). Color code: Ag, dark yellow; S, yellow; C, pale gray; O, red; N, bright green, H, dark grey.

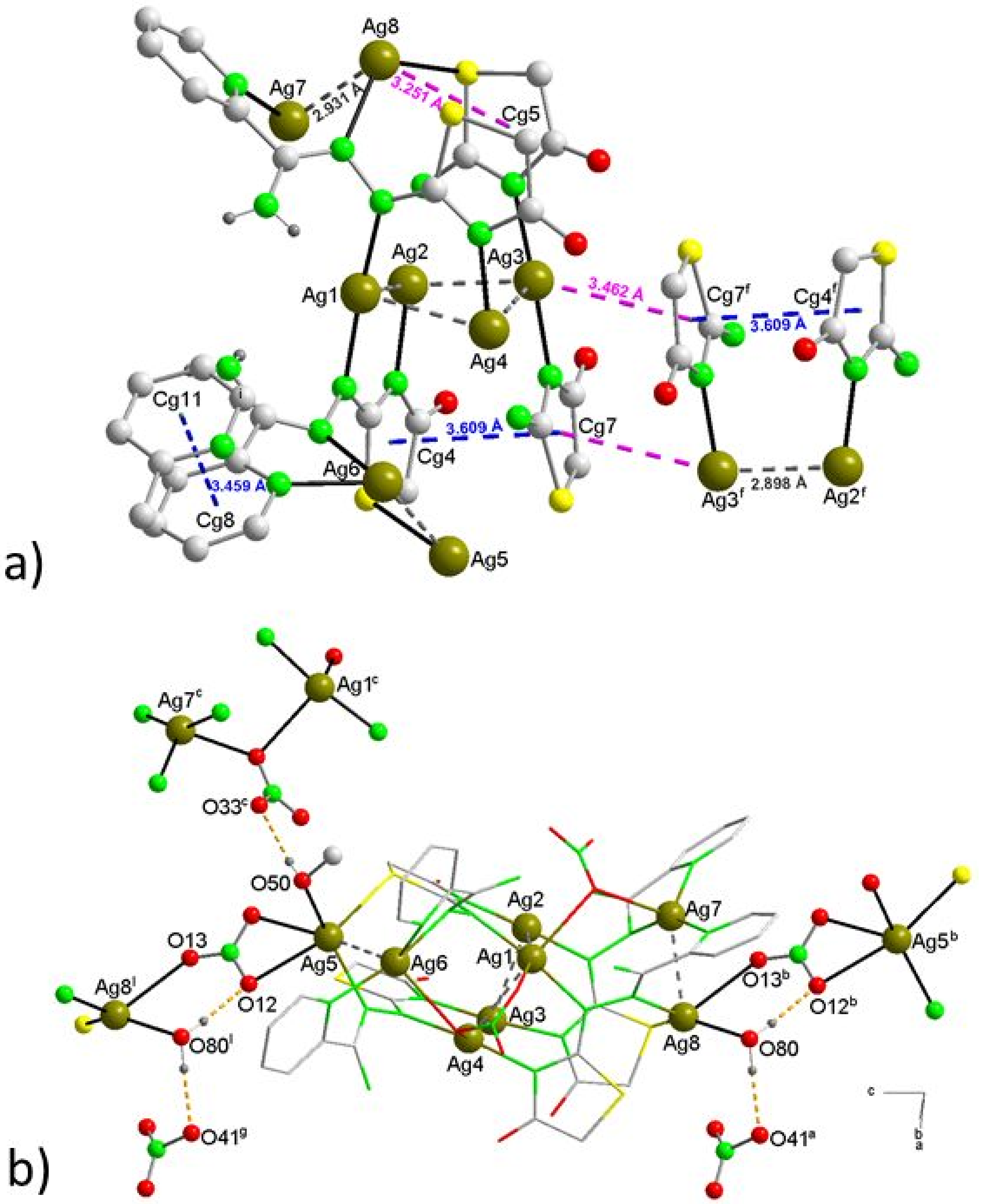

Figure S8. a) A view of silver(I)–aromatic ring and aromatic ring–aromatic ring intermolecular interactions at **1** and b) Hydrogen bond between cation and nitrate anions. The symmetry codes are as in Table S2.

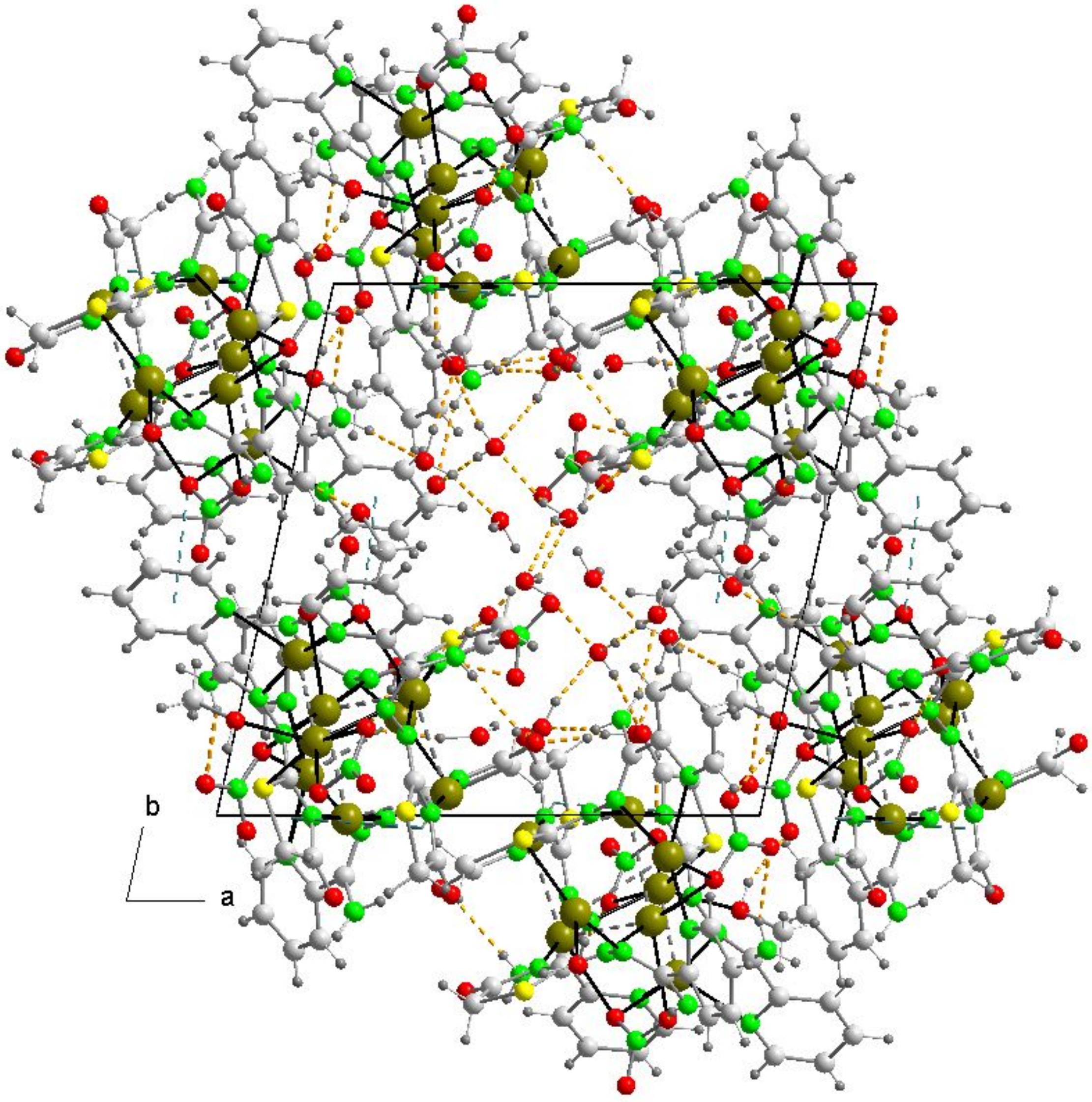

Figure S9. The crystal packing of compound **1** (along c axis). Several N-H···O and O-H···O hydrogen bonds and π···π contacts that link the components in the crystal are shown as dashed lines. Color code: Ag, dark yellow; S, yellow; C, pale gray; O, red; N, bright green, H, dark grey.

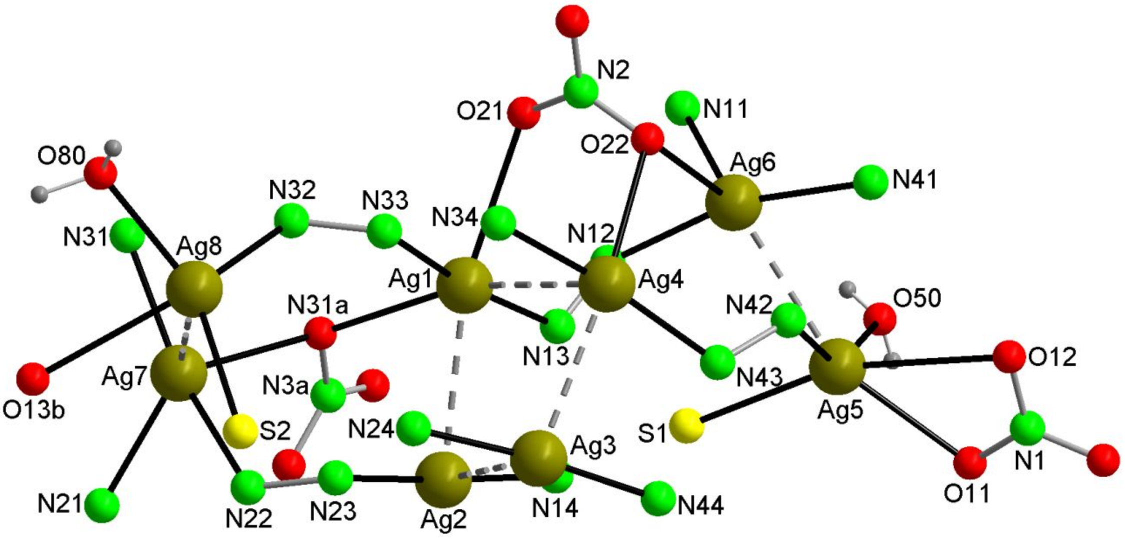

Figure S10. The cluster cation showing the coordination number and geometry in **2**.

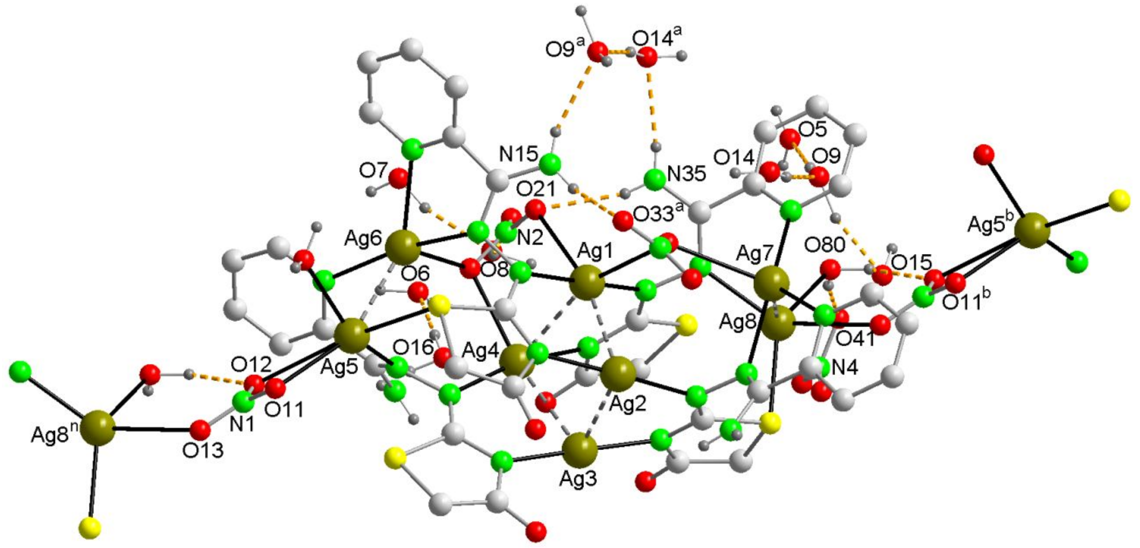

Figure S11. A view of the supramolecular packing formed in **2** via N–H···O and O–H···O interactions, with orange dashed lines representing hydrogen bonds. The symmetry codes are as in Table S3.

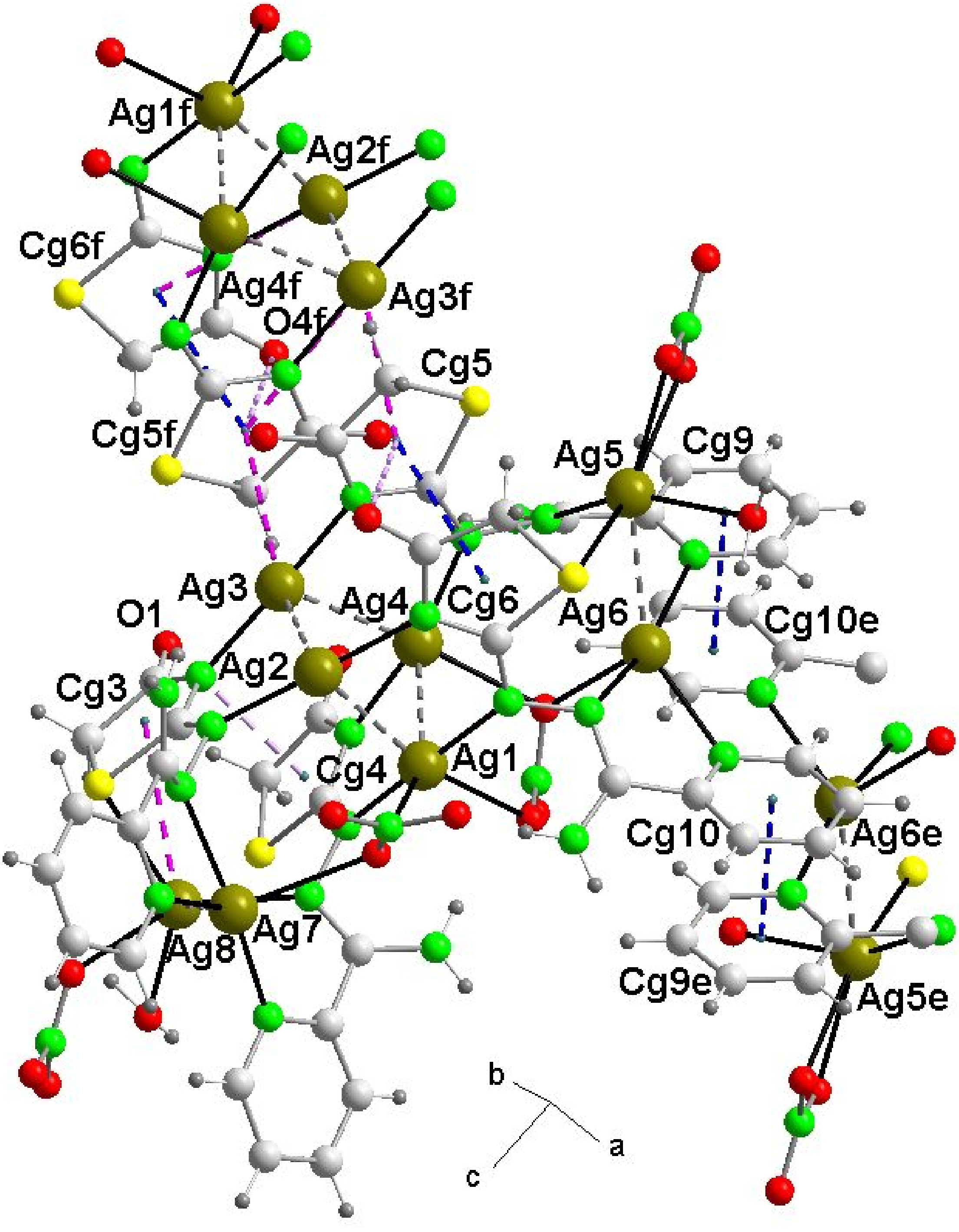

Figure S12. Representation of inter- and intramolecular π···π, ring-metal and CX-ring interactions between any neighboring molecules in **2.** The symmetry codes are as in Table S4.

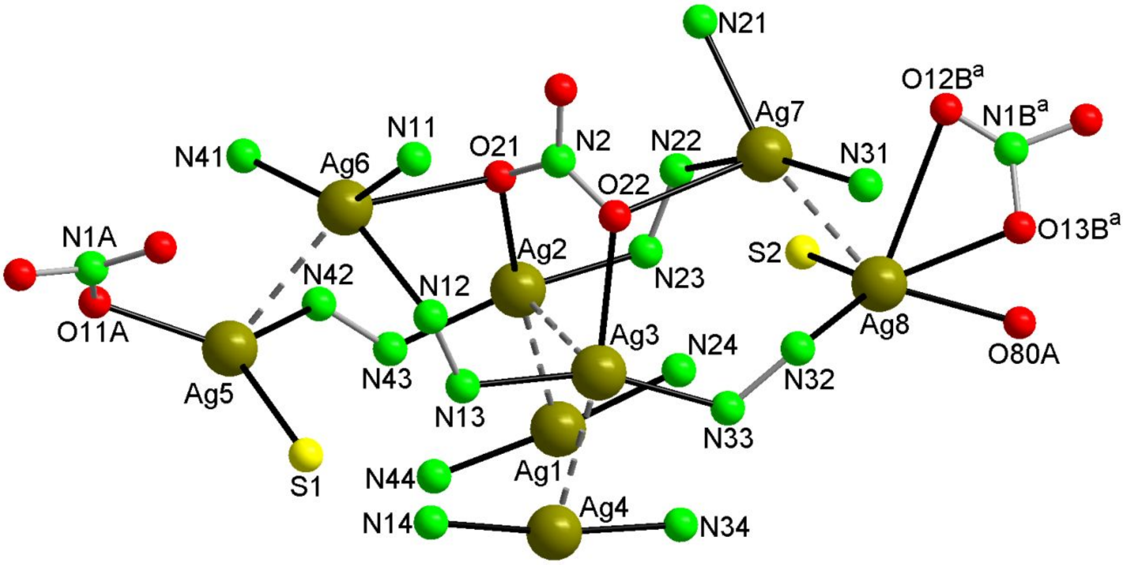

Figure S13. View of the cluster cation showing the coordination number and geometry at **3a**.

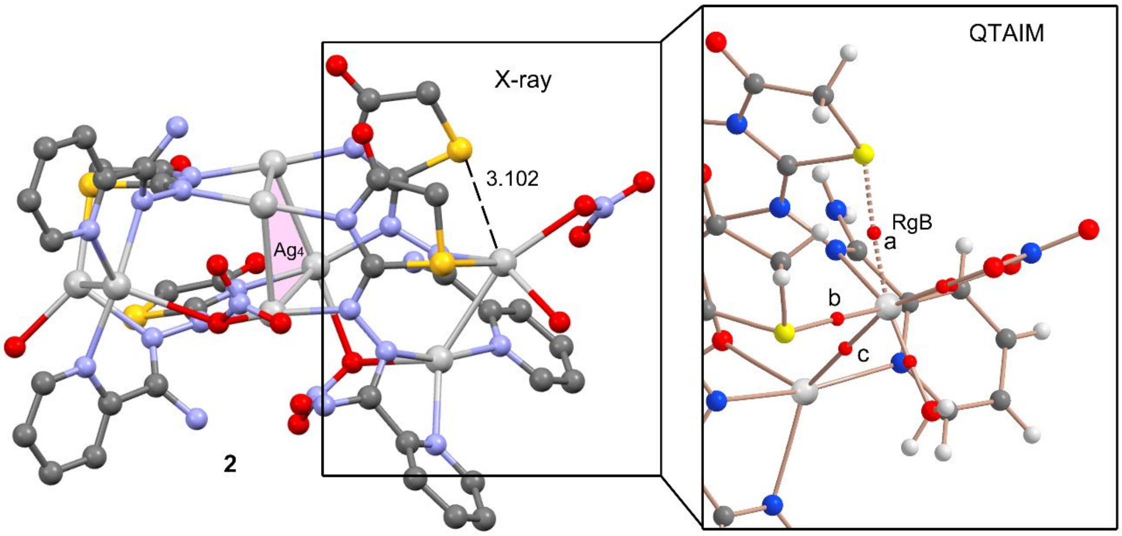

Figure S14: Left: Partial view of the X-ray structure of compound **2**. H-atoms omitted. Distance in Å. Right: Detail of the BCPs and bond paths (solid bonds) involving the Ag atom that participates in the RgB (marked as a dashed bonds).

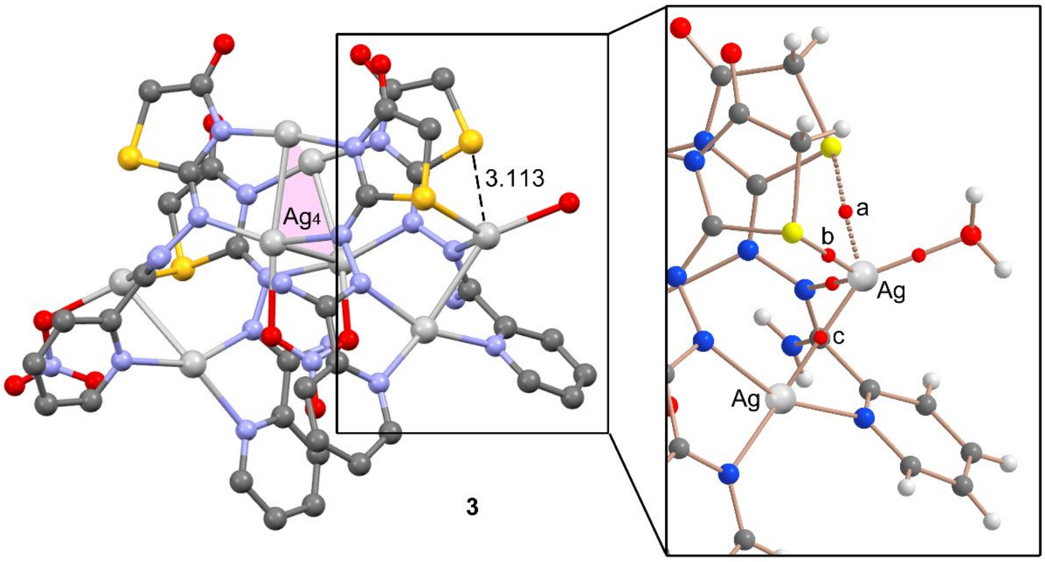

Figure S15 Left: Partial view of the X-ray structure of compound **3**. H-atoms omitted. Distance in Å. Right: Detail of the BCPs and bond paths (solid bonds) involving the Ag atom that participates in the RgB (marked as a dashed bonds).

Table S1. Hydrogen bond parameters for $[Ag_8(AmDHotaz)_4(NO_3)_3(MeOH)(H_2O)]$ $(NO_3)\cdot MeOH\cdot 7.5H_2O$ (**1**) [Å/°].

D-H···A	d(D-H)	d(H···A)	d(D···A)	∠(DHA)
O(1)-H(1A)···O(41)[a]	0.95	1.88	2.786(10)	158.2
O(1)-H(1B)···O(12)[b]	0.94	1.85	2.785(8)	168.6
O(50)-H(50A)···O(33)[c]	0.84	1.93	2.732(8)	158.6
N(15)-H(15A)···O(32)	0.84	2.19	2.987(8)	157.2
N(15)-H(15B)···O(2)[d]	0.84	2.09	2.893(8)	157.9
N(25)-H(25A)···O(6)[e]	0.84	2.20	2.983(10)	155.4
N(25)-H(25B)···O(7)[f]	0.84	2.16	2.936(11)	152.9
N(35)-H(35A)···O(60)[d]	0.84	2.07	2.840(8)	153.3
N(35)-H(35B)···O(21)	0.85	2.15	2.941(8)	154.0
N(45)-H(45A)···O(40)[f]	0.85	2.14	2.859(8)	142.6
N(45)-H(45B)···O(3)	0.84	2.13	2.940(10)	162.7
O(2)-H(2A)···O(5)	0.92	1.83	2.716(8)	161.4
O(2)-H(2B)···O(4)	0.92	1.89	2.782(12)	161.8
O(3)-H(3B)···O(9)[g]	1.02	1.99	2.763(16)	131.0
O(4)-H(4A)···O(3)[g]	0.93	2.61	3.335(14)	135.1
O(5)-H(5A)···O(11)[f]	0.94	2.23	2.956(8)	133.5
O(5)-H(5B)···O(20)	0.94	1.88	2.758(8)	156.1
O(6)-H(6B)···O(23)	0.85	2.22	2.830(10)	128.1
O(7)-H(7A)···O(40)[f]	0.83	1.95	2.778(11)	176.9
O(7)-H(7B)···O(9)[g]	0.79	1.95	2.625(17)	143.5
O(8)-H(8A)···O(7)	0.81	1.49	2.208(17)	146.0

[*)] Symmetry transformations used to generate equivalent atoms: a) -x+1, -y+1, -z; b) x, y, z-1; c) -x, -y, -z+1; d) x-1, y, z; e) x, y-1, z; f) -x+1, -y, -z+1; g) -x+1, -y+1, -z+1; h) x, y+1, z; i) -x, -y+1, -z+1; j) -x, -y, -z; k) -x+1, -y, -z.

Table S2. Intermolecular ring-ring, ring-metal and CX-ring interaction parameters (Å, º) for **1***.

Comp.	Ring	π···π	Cg-Cg	α	IPD	Symmetry
**1**	S1/C17/N14/C19/C18<br>S4/C47/N44/C49/C48	Cg4-Cg7	3.609	13.59	3.319	
	N11/C11/C12/C13/C14/C15<br>N41/C41/C42/C43/C44/C45	Cg8-Cg11[i]	3.459	6.29	3.427	-x,1-y,1-z
	S2/C27/N24/C29/C28	Cg5-Ag8	3.251	41.79	2.424	
	S4/C47/N44/C49/C48	Cg7-Ag3[f]	3.462	22.19	3.206	1-x, -y, 1-z
	Ag5/O11/N1/O12	C31-H31···Cg2	2.960	26.77	-2.640	
	N11/C11/C12/C13/C14/C15	C51-H51A···Cg8[b]	2.990	21.46	2.780	x, y, -1+z
	S3/C37/N34/C39/C38	C29-O20···Cg6	3.281	21.98	-3.042	

*CgI/CgJ are the centroids of the corresponding rings. Cg-Cg is the center-to-center distance (distance between ring centroids), α is the angle between mean planes of the rings, IPD is the mean interplanar distance (distance from one plane to the neighboring centroid) and SA is the mean slippage angle (angle subtended by the intercentroid vector to the plane normal). For details, see Janiak, C. (2000). J. Chem. Soc. Dalton Trans. pp. 3885–3898.

Table S3. Hydrogen bond parameters for {[Ag_8(AmDHotaz)$_4$(NO_3)$_3$(H_2O)$_2$](NO_3)·9.5(H_2O)}$_n$ (**2**) [Å/ °].

D-H···A	d(D-H)	d(H···A)	d(D···A)	∠(DHA)
O(50)-H(50A)···O(7)^c	0.85	1.84	2.683(5)	169.1
O(50)-H(50B)···O(32)^d	0.79	2.02	2.805(5)	173.2
O(80)-H(80A)···O(41)	0.98	1.75	2.717(9)	168.4
O(80)-H(80A)···O(42)	0.98	2.57	3.209(10)	122.6
O(80)-H(80A)···N(4)	0.98	2.47	3.349(14)	149.9
O(80)-H(80B)···O(12)^b	0.98	1.99	2.927(7)	158.5
N(15)-H(15A)···O(31)^a	0.85	2.61	3.395(5)	155.0
N(15)-H(15A)···O(33)^a	0.85	2.30	3.039(5)	145.8
N(15)-H(15B)···O(9)^a	0.79	2.19	2.934(6)	157.3
N(25)-H(25A)···O(6)^e	0.81	2.16	2.914(6)	156.7
N(25)-H(25B)···O(8)^e	0.90	2.13	2.961(6)	153.3
N(35)-H(35A)···O(21)	0.81	2.18	2.965(5)	161.9
N(35)-H(35B)···O(14)^a	0.80	2.17	2.961(7)	171.7
N(45)-H(45A)···O(3)f	0.83	2.01	2.801(5)	157.7
N(45)-H(45B)···O(17)^c	0.79	2.20	2.901(11)	149.1
C(11)-H(11)···O(22)^c	0.95	2.47	3.211(6)	134.8
C(18)-H(18A)···N(15)^g	0.99	2.60	3.431(6)	141.4
C(18)-H(18B)···S(4)	0.99	2.96	3.695(5)	131.5
C(22)-H(22)···O(31)^e	0.95	2.54	3.294(6)	136.4
C(24)-H(24)···O(43)^h	0.95	2.60	3.474(13)	152.3
C(28)-H(28A)···O(42)	0.99	2.43	3.294(8)	145.6
C(28)-H(28B)···O(13)^f	0.99	2.49	3.448(6)	163.2
C(32)-H(32)···O(50)^b	0.95	2.49	3.242(6)	136.3
C(38)-H(38A)···O(43)^i	0.99	2.64	3.617(14)	170.0

Symmetry transformations used to generate equivalent atoms: a) -x+1,-y+1,-z+1; b) x,y,z+1; c) -x+1,-y+1,-z; d) x,y+1,z-1; e) x,y+1,z; f) -x,-y+2,-z; g) -x+1,-y+2,-z; h) -x,-y+2,-z+1; i) -x,-y+1,-z+1

Table S4. Intermolecular ring-ring, ring-metal and CX-ring interaction parameters (Å, º) for **2***.

**Comp.**	**Ring**	**π···π**	**Cg-Cg**	**α**	**IPD**	**Symmetry**
**2**	S1/C17/N14/C19/C18<br>S4/C47/N44/C49/C48	Cg3-Cg6	3.607	13.81	3.509	
	N11/C11/C12/C13/C14/C15<br>N41/C41/C42/C43/C44/C45	Cg7-Cg10^i	3.460	4.15	3.412	1-x,1-y, -z
	S2/C27/N24/C29/C28	Cg4-Ag8	3.266	41.39	-2.450	
	S4/C47/N44/C49/C48	Cg6-Ag3^f	3.449	22.29	-3.191	-x, 2-y, -z
	S3/C37/N34/C39/C38	C29-O4···Cg5	3.324	20.96	3.104	
	N41/C41/C42/C43/C44/C45	N1-O12···Cg10	3.270	17.06	-3.126	

*CgI/CgJ are the centroids of the corresponding rings. Cg-Cg is the center-to-center distance (distance between ring centroids), α is the angle between mean planes of the rings, IPD is the mean interplanar distance (distance from one plane to the neighboring centroid) and SA is the mean slippage angle (angle subtended by the intercentroid vector to the plane normal). For details, see Janiak, C. (2000). J. Chem. Soc. Dalton Trans. pp. 3885–3898.

Table S5. Hydrogen bond parameters for $\{[Ag_8(AmDHotaz)_4(NO_3)_2(H_2O)](NO_3)(OH)\cdot 4.5H_2O\}_n$ (**3**) [Å/ °].

D-H···A	d(D-H)	d(H···A)	d(D···A)	∠(DHA)
N(15)-H(15A)...O(6)[c]	0.88	2.13	2.844(17)	137.7
N(15)-H(15B)...O(31)[c]	0.88	1.96	2.761(17)	150.7
N(25)-H(25A)...O(5)[c]	0.88	2.13	2.974(18)	159.4
N(25)-H(25B)...O(32)[d]	0.88	2.65	3.319(19)	134.1
N(25)-H(25B)...O(33)[d]	0.88	2.10	2.941(19)	160.8
N(35)-H(35A)...O(4)[e]	0.88	2.23	2.927(18)	136.3
N(35)-H(35B)...O(31)[c]	0.88	2.36	3.02(2)	131.6
N(35)-H(35B)...O(32)[c]	0.88	2.56	3.42(2)	165.8
N(45)-H(45B)...O(7)[d]	0.88	2.06	2.860(19)	150.8
O(5)-H(5A)...O(1)[g]	0.96	1.96	2.913(17)	173.9
O(5)-H(5B)...O(3)[g]	0.96	1.91	2.805(19)	154.2
O(6)-H(6A)...O(4)[h]	0.96	1.92	2.812(19)	154.8
O(6)-H(6B)...O(2)[h]	0.96	1.92	2.824(18)	156.7
O(7)-H(7A)...O(31)	0.96	2.59	3.437(19)	147.2
O(7)-H(7A)...O(33)	0.96	1.90	2.798(19)	155.3
O(7)-H(7B)...O(6)	0.96	2.02	2.97(2)	177.1
C(11)-H(11)...O(32)	0.95	2.36	2.96(2)	121.1
C(12)-H(12)...O(9)	0.95	2.59	3.49(3)	160.2
C(14)-H(14)...O(31)[c]	0.95	2.54	3.38(2)	147.5
C(21)-H(21)...O(7)[a]	0.95	2.44	3.23(2)	141.0
C(24)-H(24)...O(33)[d]	0.95	2.56	3.48(2)	162.2
C(31)-H(31)...O(33)[a]	0.95	2.58	3.29(2)	132.2
C(39)-H(39A)...O(2)	0.99	2.44	3.24(2)	136.7
C(41)-H(41)...O(31)	0.95	2.46	3.18(2)	132.3
C(41)-H(41)...O(32)	0.95	2.45	3.39(2)	173.3
C(49)-H(49A)...O(1)	0.99	2.64	3.38(2)	131.7
C(49)-H(49B)...S(3)[f]	0.99	2.98	3.700(18)	130.6

Symmetry transformations used to generate equivalent atoms: a) x,y-1,z; b) x,y+1,z; c) -x+1/2,y-1/2,z-1/2; d) -x+1/2,y-1/2,z+1/2; e) -x+1,-y,z-1/2; f) -x+1,-y,z+1/2; g) x-1/2,-y+1/2,z+1; h) x-1/2,-y+1/2,z; i) x,y,-1+z

Table S6. Intermolecular ring-ring, ring-metal and CX-ring interaction parameters (Å, °) for **3***.

**Comp.**	**Ring**	**π···π**	**Cg-Cg**	**α**	**IPD**	**Symmetry**
**3**	S2/C27/N24/C29/C28<br>S3/C37/N34/C39/C38	Cg4-Cg5	3.765	10.97	3.266	
	S3/C37/N34/C39/C38<br>S4/C47/N44/C49/C48	Cg5-Cg6[e]	3.713	9.05	3.667	1-x,-y,-1/2+z
	N11/C11/C12/C13/C14/C15<br>N21/C21/C22/C23/C24/C25	Cg7-Cg8[i]	3.608	3.12	3.306	1/2-x,1/2+y,-1/2+z
	S1/C17/N14/C19/C18	Cg3-Ag5	3.34	42.69	2.457	
	S2/C27/N24/C29/C28	Cg4-Ag8	3.37	42.68	2.480	
	Ag5/O11/N1/O12	N2-O23···Cg2	3.31	21.11	3.088	
	S1/C17/N14/C19/C18	C28-O2···Cg3[e]	3.28	19.12	-3.100	1-x,-y,1/2+z

*CgI/CgJ are the centroids of the corresponding rings. Cg-Cg is the center-to-center distance (distance between ring centroids), α is the angle between mean planes of the rings, IPD is the mean interplanar distance (distance from one plane to the neighboring centroid) and SA is the mean slippage angle (angle subtended by the intercentroid vector to the plane normal). For details, see Janiak, C. (2000). J. Chem. Soc. Dalton Trans. pp. 3885–3898.
